# Supplementary figures and images for: Association with TFIIIC limits MYCN localisation in hubs of active promoters and chromatin accumulation of non-phosphorylated RNA polymerase II
Source: eLife. 2024 Aug 23;13:RP94407. doi: 10.7554/eLife.94407 (PMC11343564; doi:10.7554/eLife.94407)

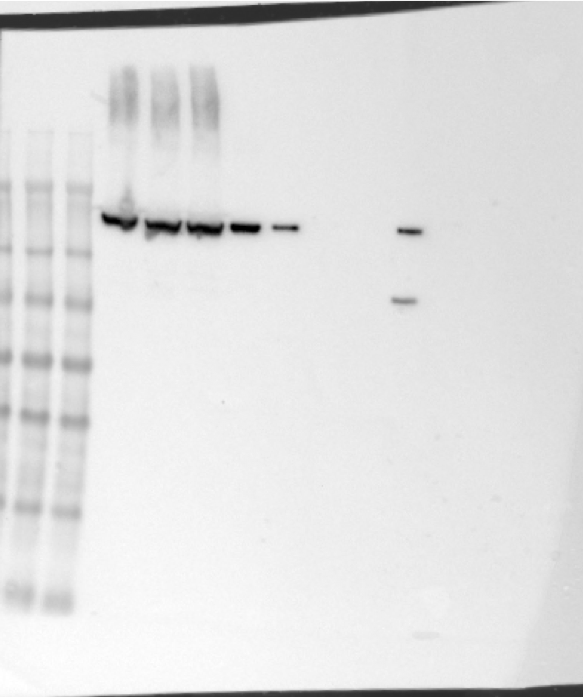

Supplement: Figure 1—source data 1. [file elife-94407-fig1-data1.zip › Figure 1 - source data 1/1A-WBs raw images /TFIIIC4-WB.tiff]

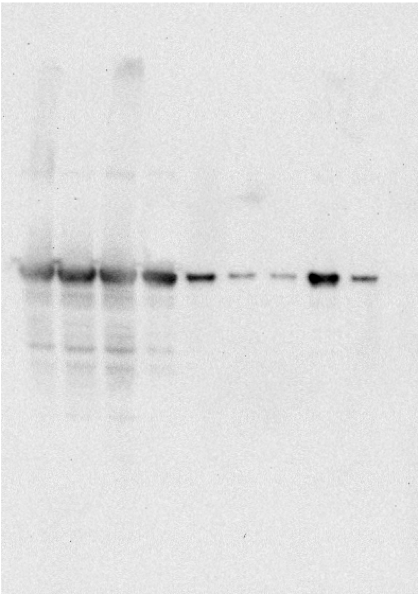

Supplement: Figure 1—source data 1. [file elife-94407-fig1-data1.zip › Figure 1 - source data 1/1A-WBs raw images /TFIIIC5-WB.tiff]

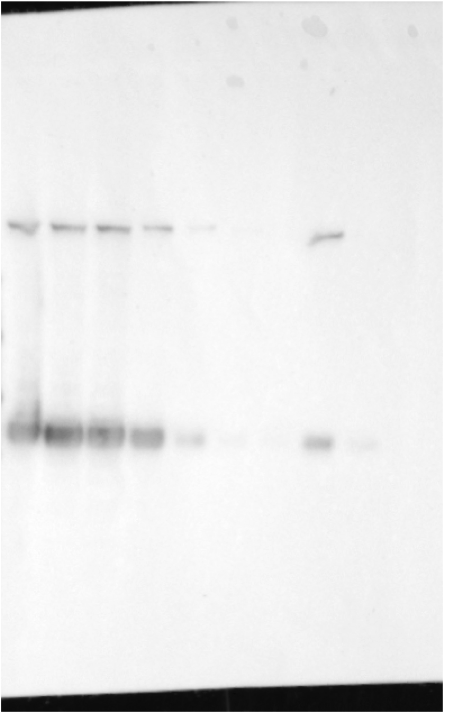

Supplement: Figure 1—source data 1. [file elife-94407-fig1-data1.zip › Figure 1 - source data 1/1A-WBs raw images /TFIIIC6-WB.tiff]

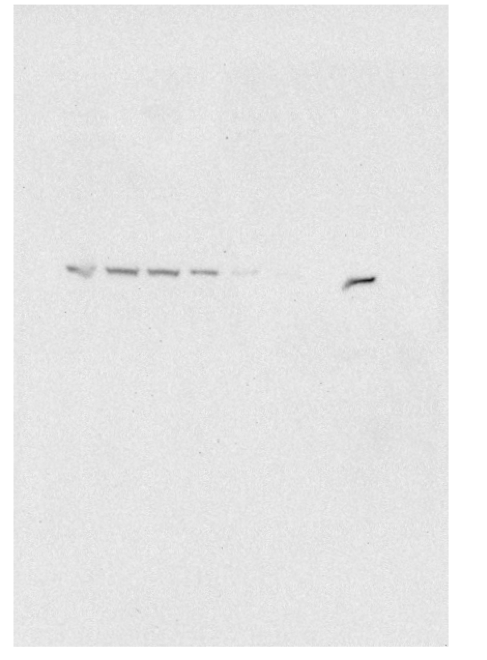

Supplement: Figure 1—source data 1. [file elife-94407-fig1-data1.zip › Figure 1 - source data 1/1A-WBs raw images /TFIIIC3-WB.tiff]

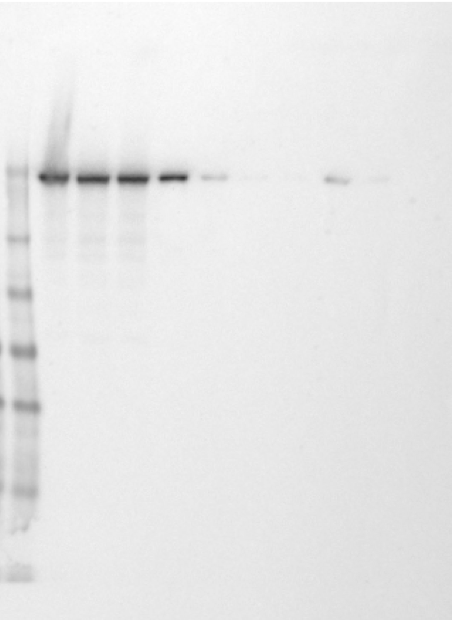

Supplement: Figure 1—source data 1. [file elife-94407-fig1-data1.zip › Figure 1 - source data 1/1A-WBs raw images /TFIIIC2-WB.tiff]

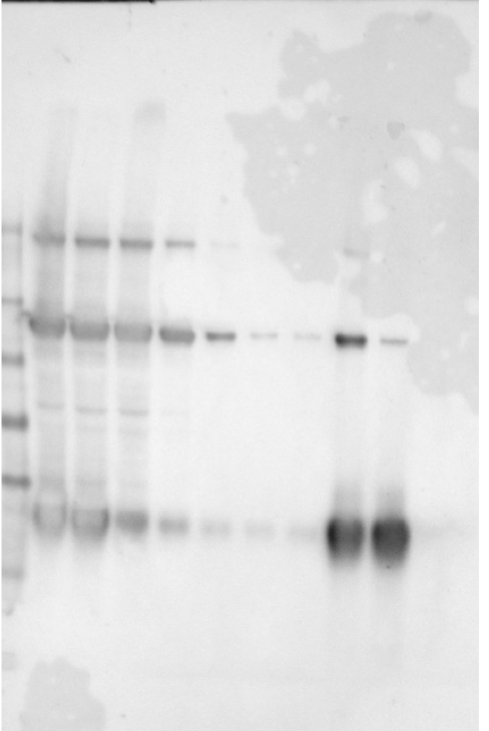

Supplement: Figure 1—source data 1. [file elife-94407-fig1-data1.zip › Figure 1 - source data 1/1A-WBs raw images /Nmyc-WB.tiff]

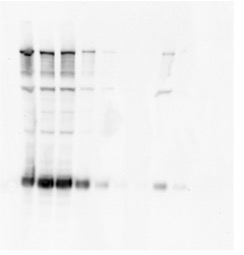

Supplement: Figure 1—source data 1. [file elife-94407-fig1-data1.zip › Figure 1 - source data 1/1A-WBs raw images /TFIIIC1-WB.tiff]

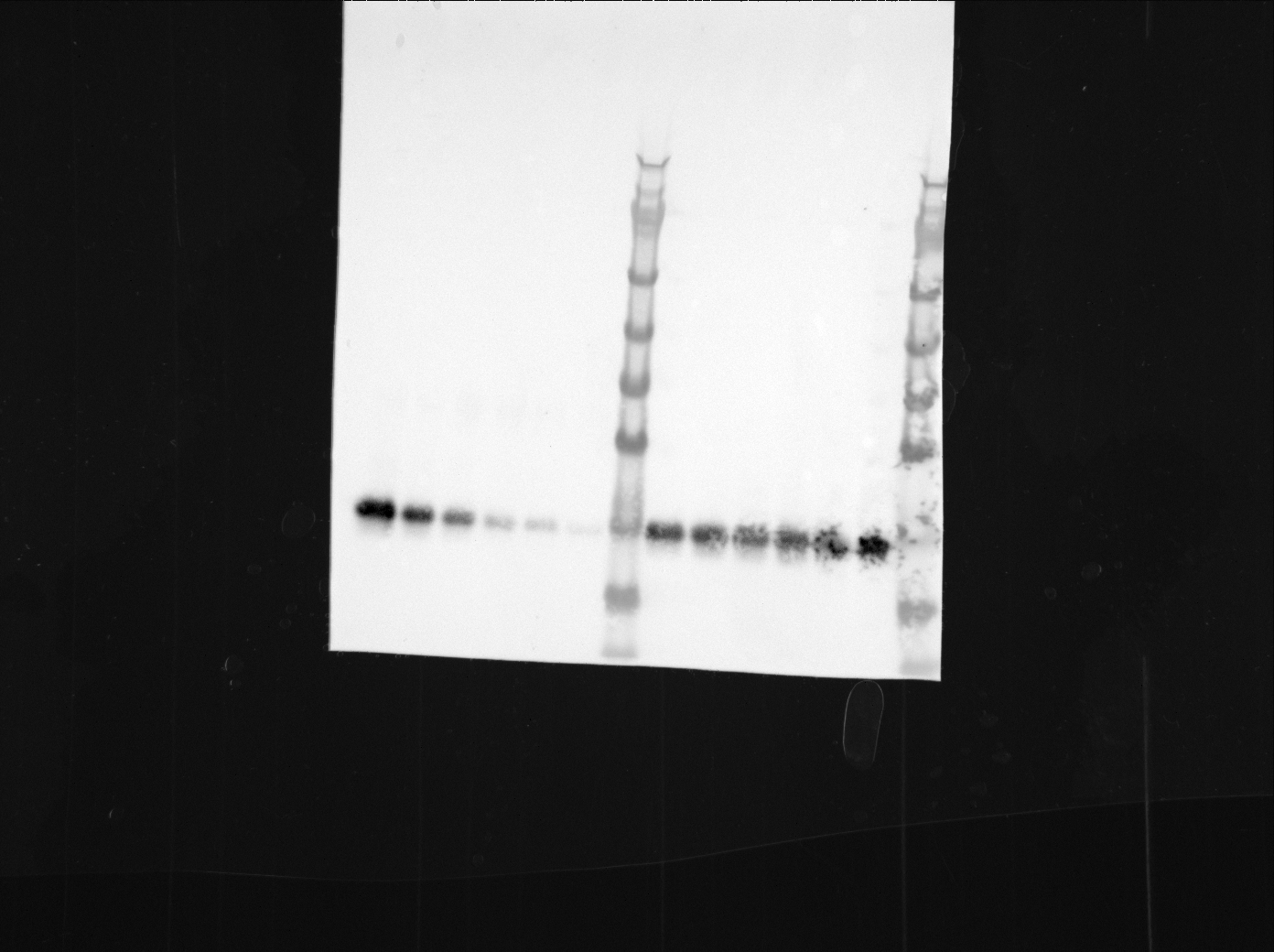

Supplement: Figure 1—source data 1. [file elife-94407-fig1-data1.zip › Figure 1 - source data 1/1D-WBs raw images/1st-anti-nmyc.tif]

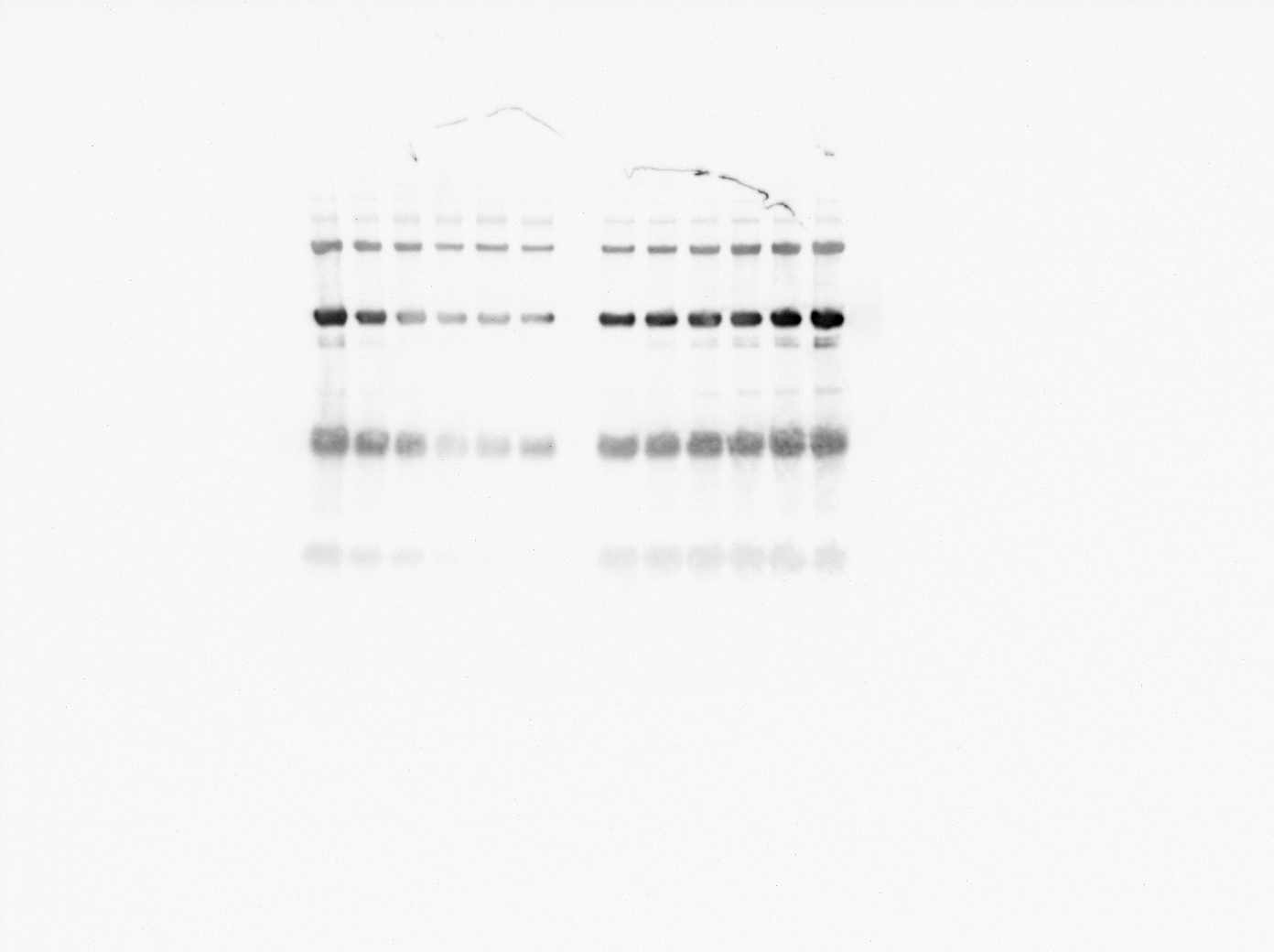

Supplement: Figure 1—source data 1. [file elife-94407-fig1-data1.zip › Figure 1 - source data 1/1D-WBs raw images/4th-anti-nmyc-anti-35-anti-102-anti-63.tif]

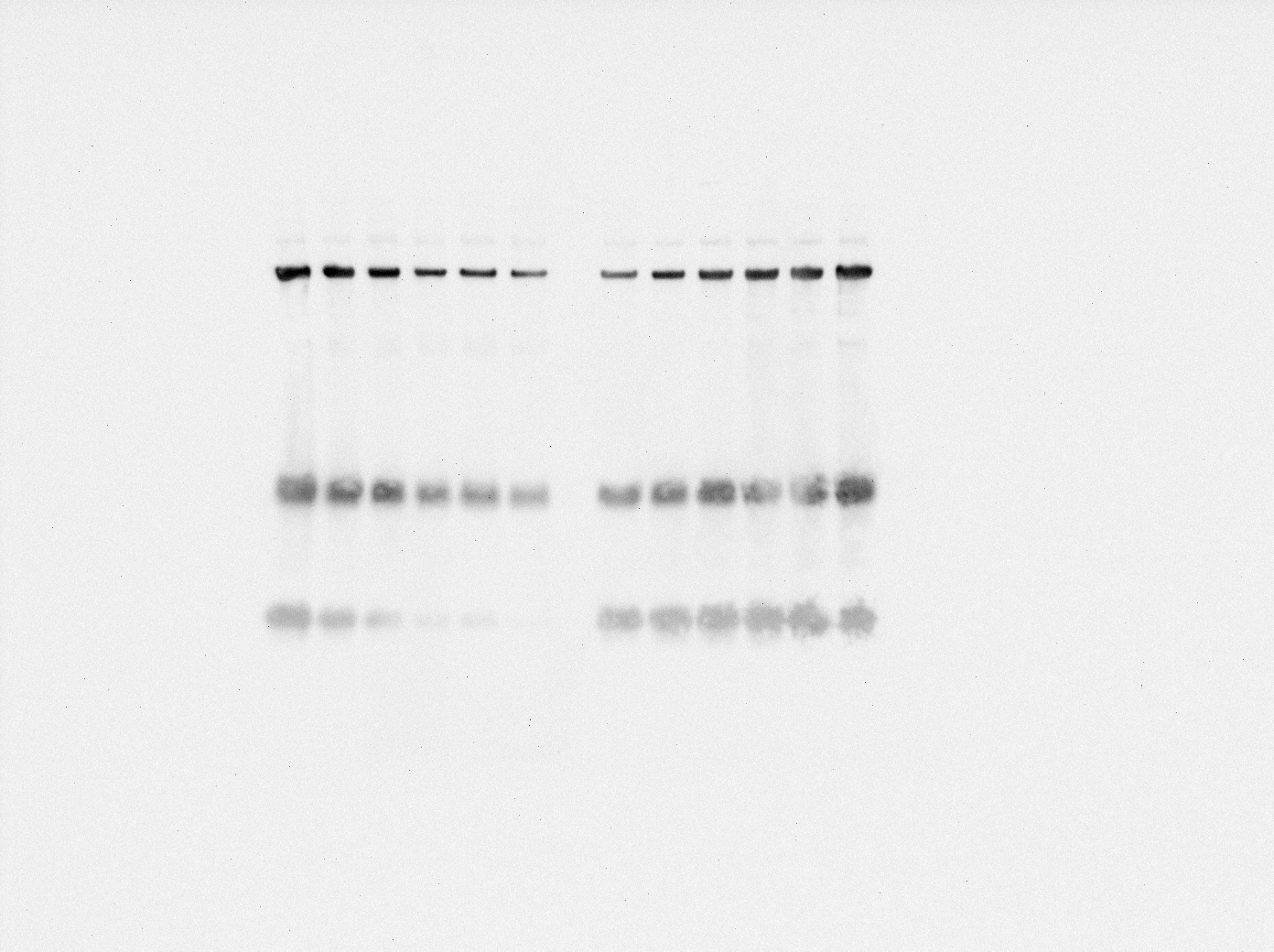

Supplement: Figure 1—source data 1. [file elife-94407-fig1-data1.zip › Figure 1 - source data 1/1D-WBs raw images/3d-anti-nmyc-anti-35-anti-102.tif]

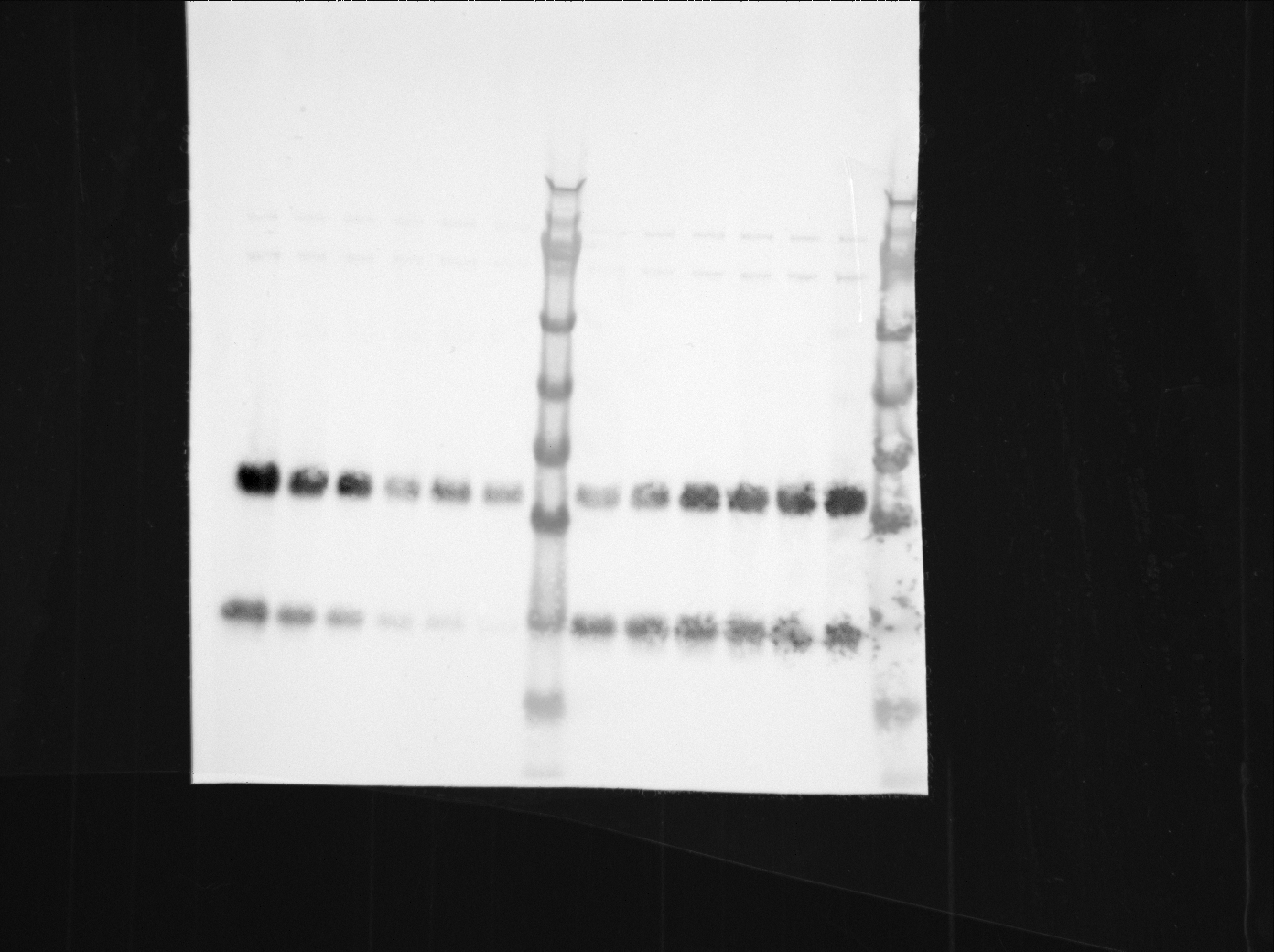

Supplement: Figure 1—source data 1. [file elife-94407-fig1-data1.zip › Figure 1 - source data 1/1D-WBs raw images/2nd-anti-nmyc-anti-35.tif]

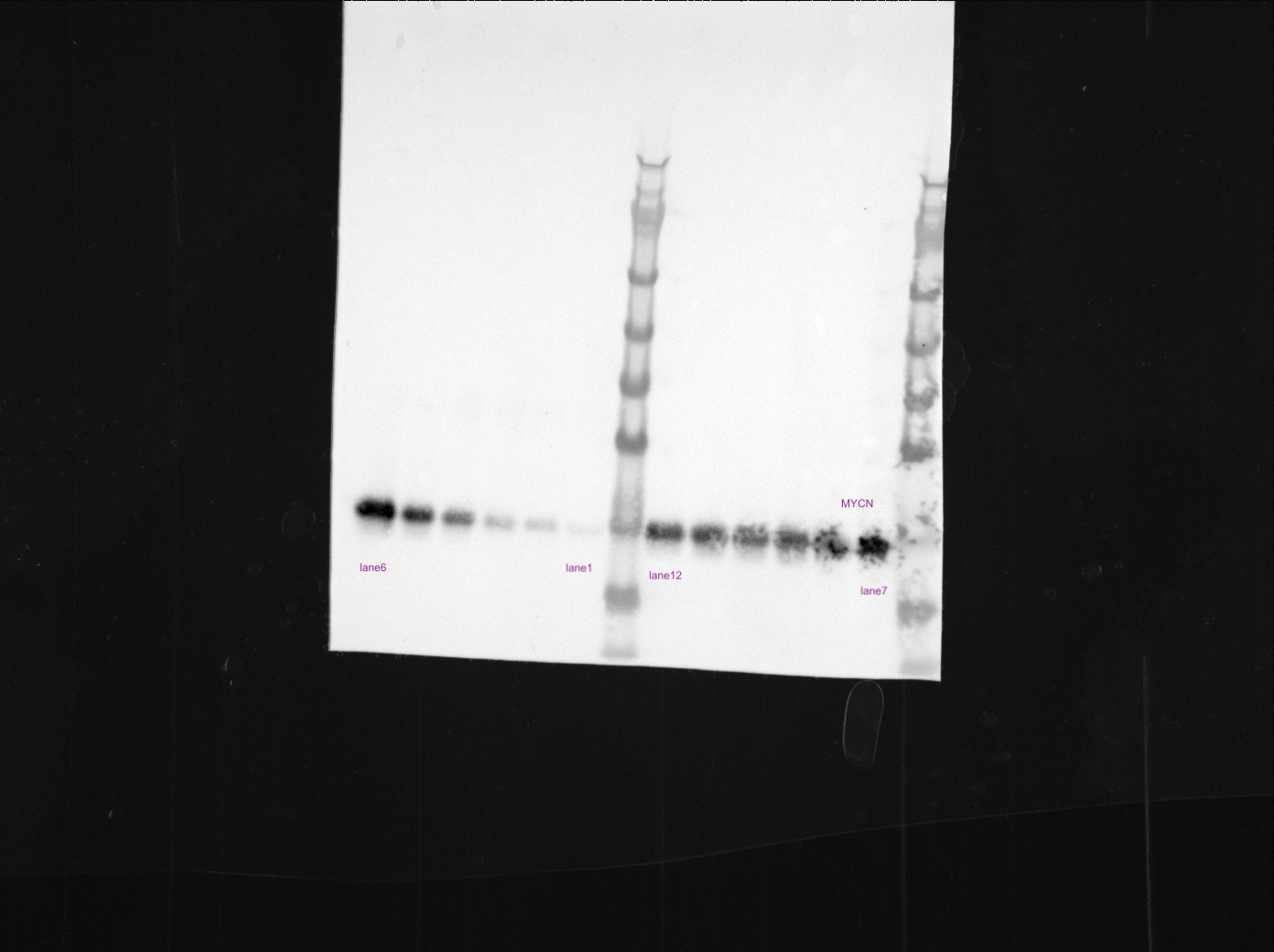

Supplement: Figure 1—source data 2. [file elife-94407-fig1-data2.zip › Figure 1 - source data 2/1D-WBs raw images labelled/1st-anti-nmyc.tif]

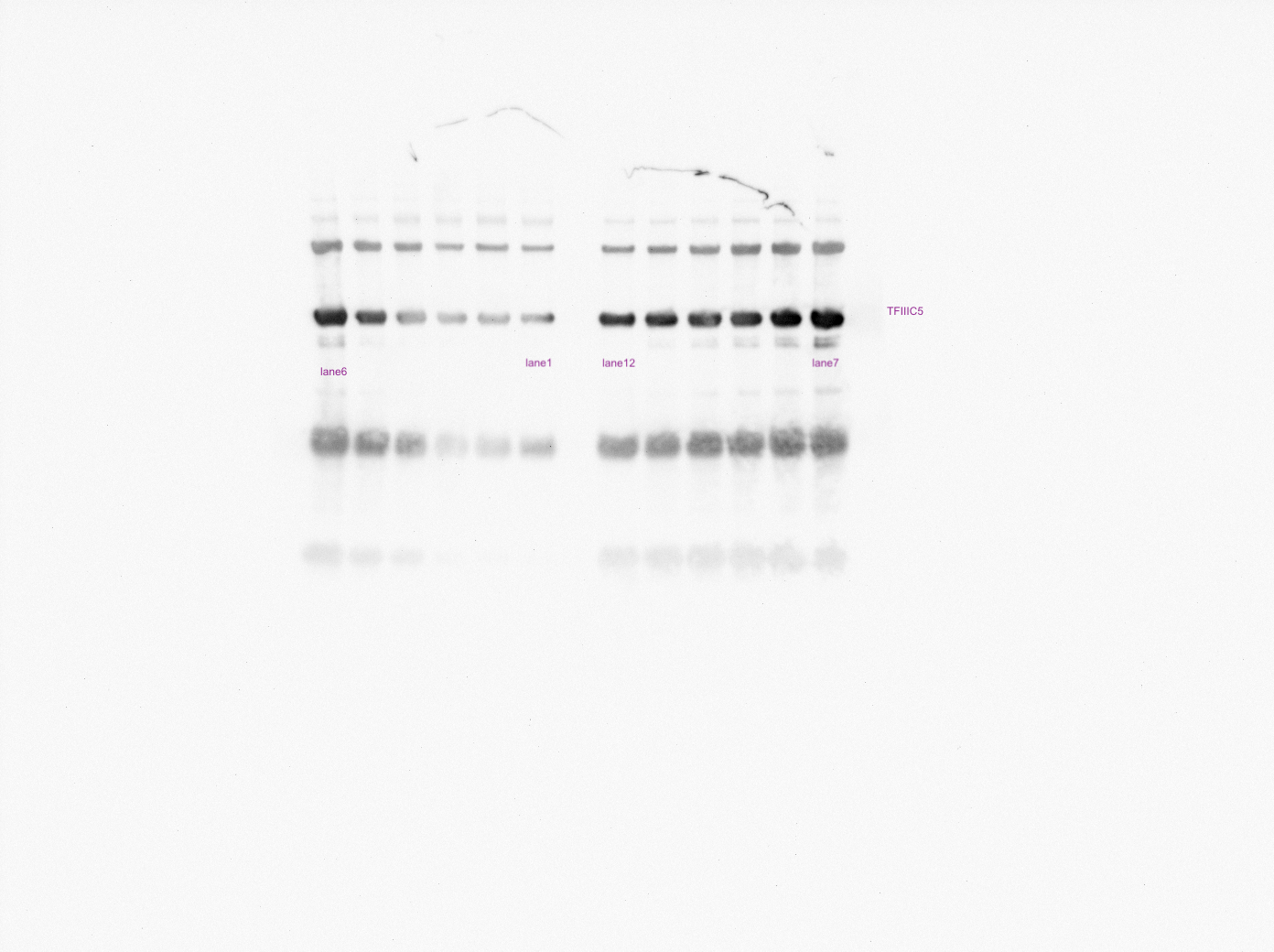

Supplement: Figure 1—source data 2. [file elife-94407-fig1-data2.zip › Figure 1 - source data 2/1D-WBs raw images labelled/4th-anti-nmyc-anti-35-anti-102-anti-63.tif]

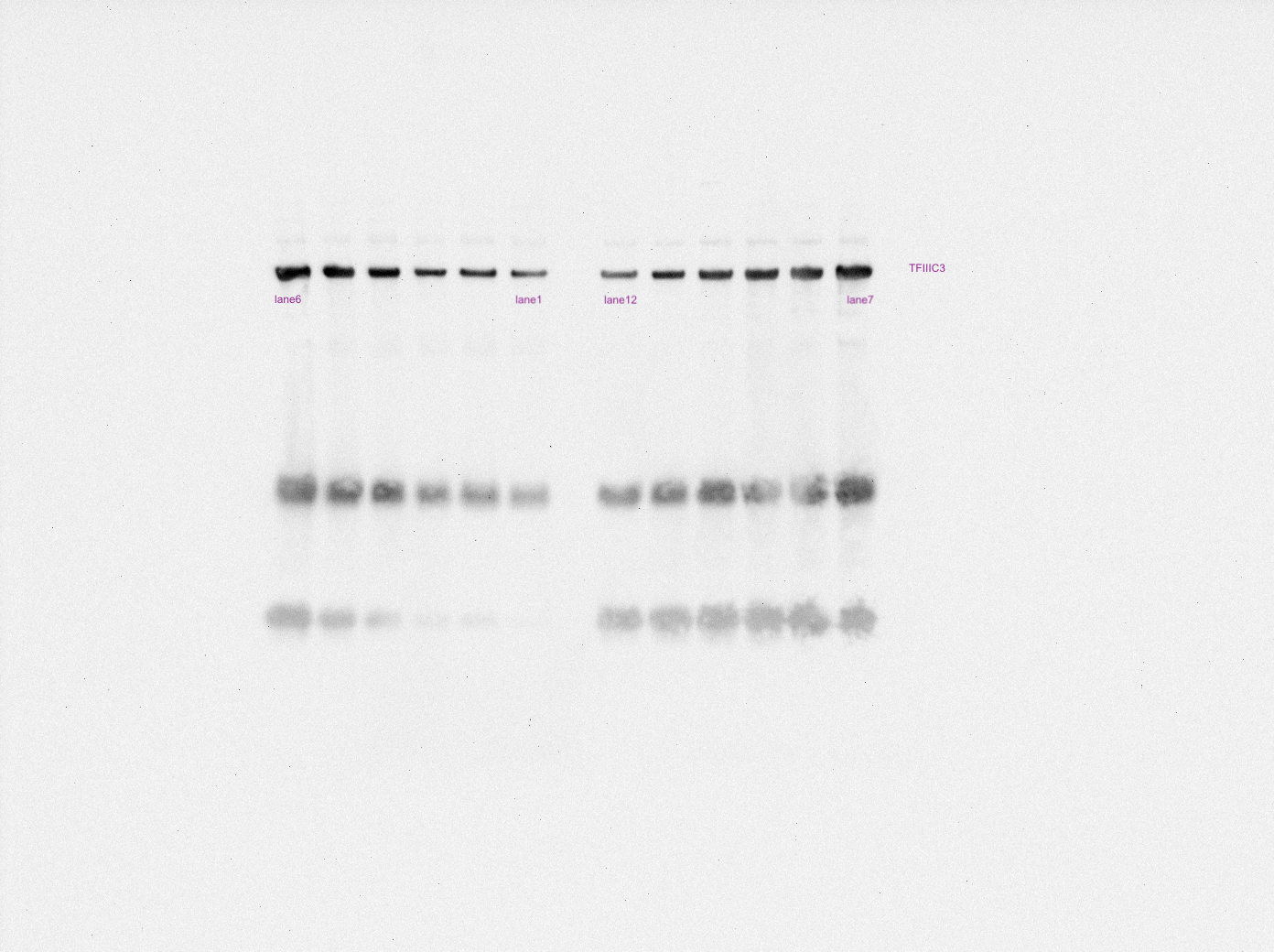

Supplement: Figure 1—source data 2. [file elife-94407-fig1-data2.zip › Figure 1 - source data 2/1D-WBs raw images labelled/3d-anti-nmyc-anti-35-anti-102.tif]

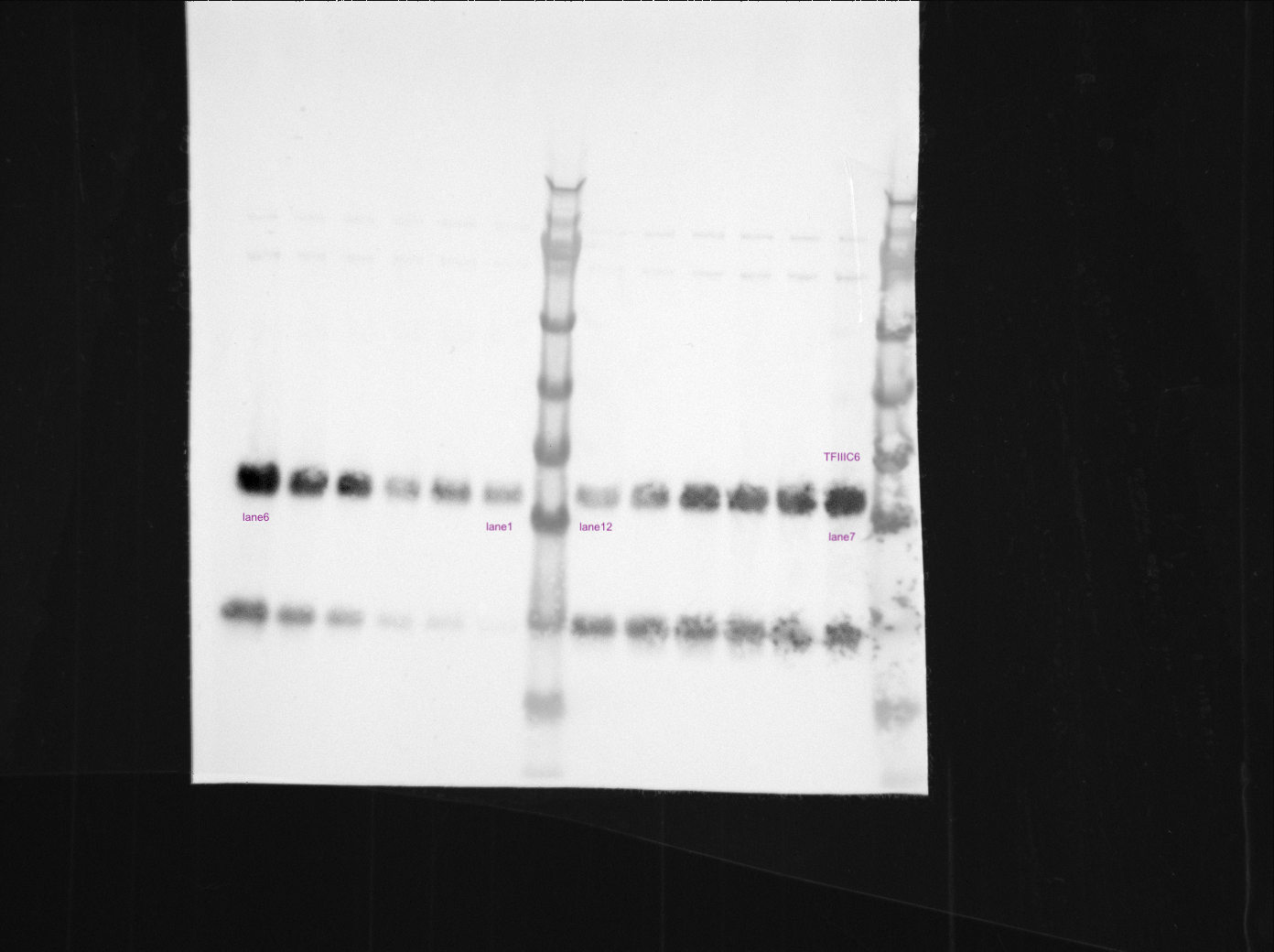

Supplement: Figure 1—source data 2. [file elife-94407-fig1-data2.zip › Figure 1 - source data 2/1D-WBs raw images labelled/2nd-anti-nmyc-anti-35.tif]

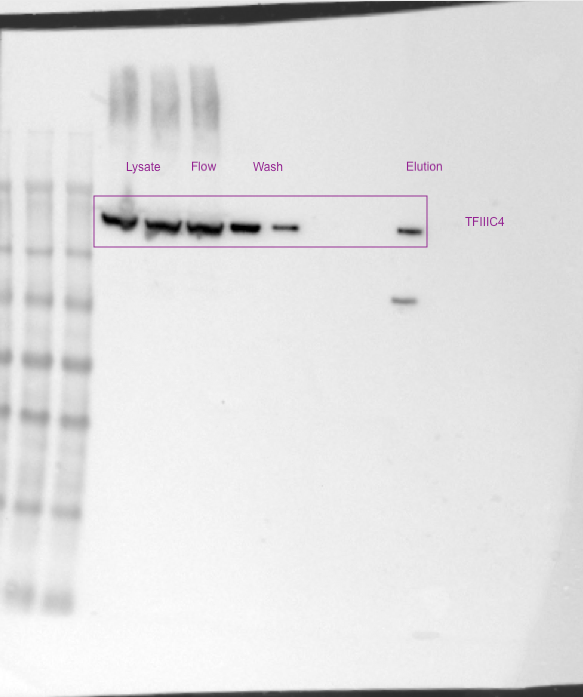

Supplement: Figure 1—source data 2. [file elife-94407-fig1-data2.zip › Figure 1 - source data 2/1A-WBs raw images labelled/TFIIIC4-WB.tiff]

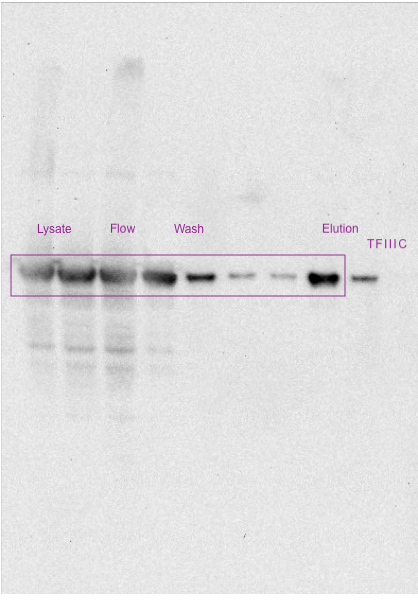

Supplement: Figure 1—source data 2. [file elife-94407-fig1-data2.zip › Figure 1 - source data 2/1A-WBs raw images labelled/TFIIIC5-WB.tiff]

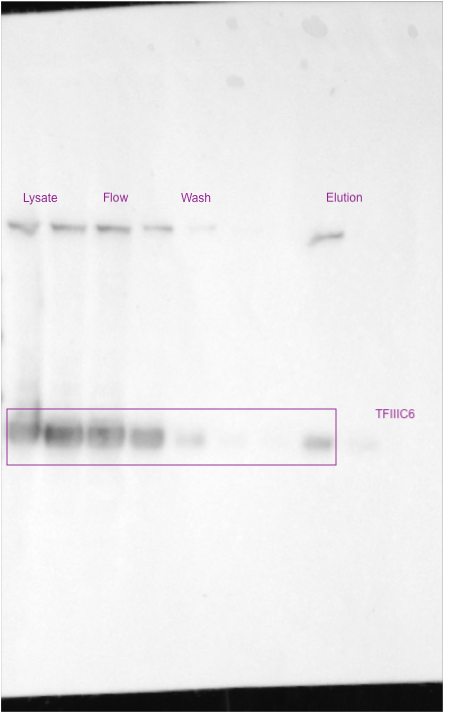

Supplement: Figure 1—source data 2. [file elife-94407-fig1-data2.zip › Figure 1 - source data 2/1A-WBs raw images labelled/TFIIIC6-WB.tiff]

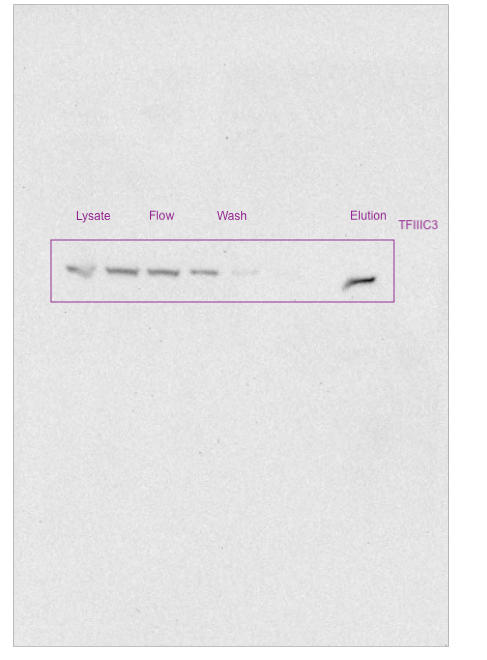

Supplement: Figure 1—source data 2. [file elife-94407-fig1-data2.zip › Figure 1 - source data 2/1A-WBs raw images labelled/TFIIIC3-WB.tiff]

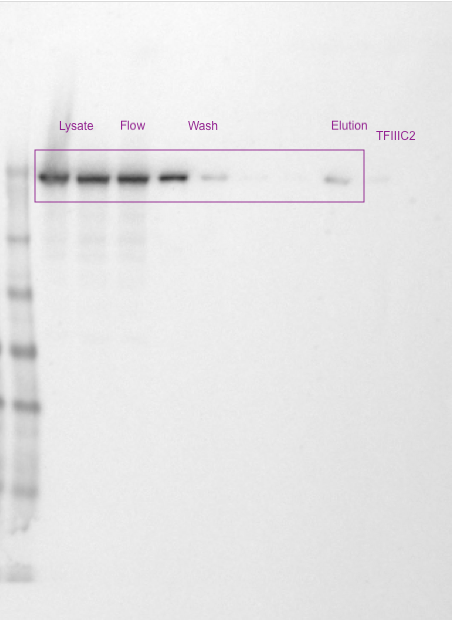

Supplement: Figure 1—source data 2. [file elife-94407-fig1-data2.zip › Figure 1 - source data 2/1A-WBs raw images labelled/TFIIIC2-WB.tiff]

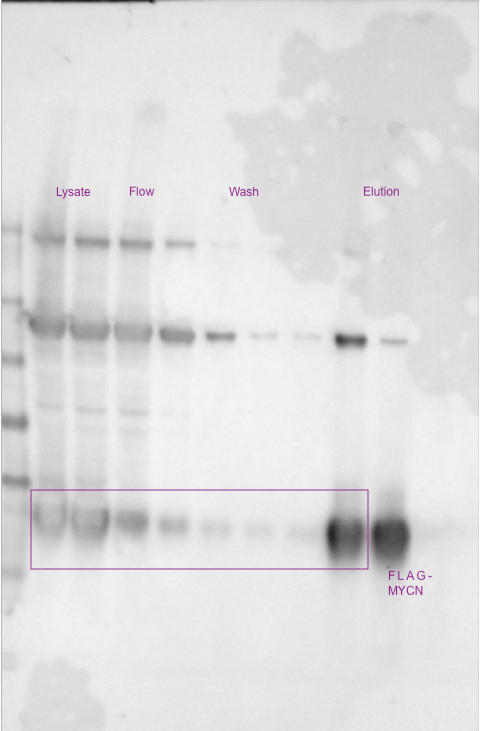

Supplement: Figure 1—source data 2. [file elife-94407-fig1-data2.zip › Figure 1 - source data 2/1A-WBs raw images labelled/Nmyc-WB.tiff]

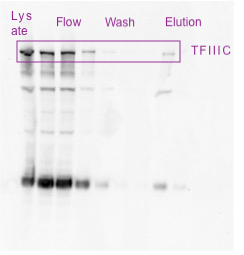

Supplement: Figure 1—source data 2. [file elife-94407-fig1-data2.zip › Figure 1 - source data 2/1A-WBs raw images labelled/TFIIIC1-WB.tiff]

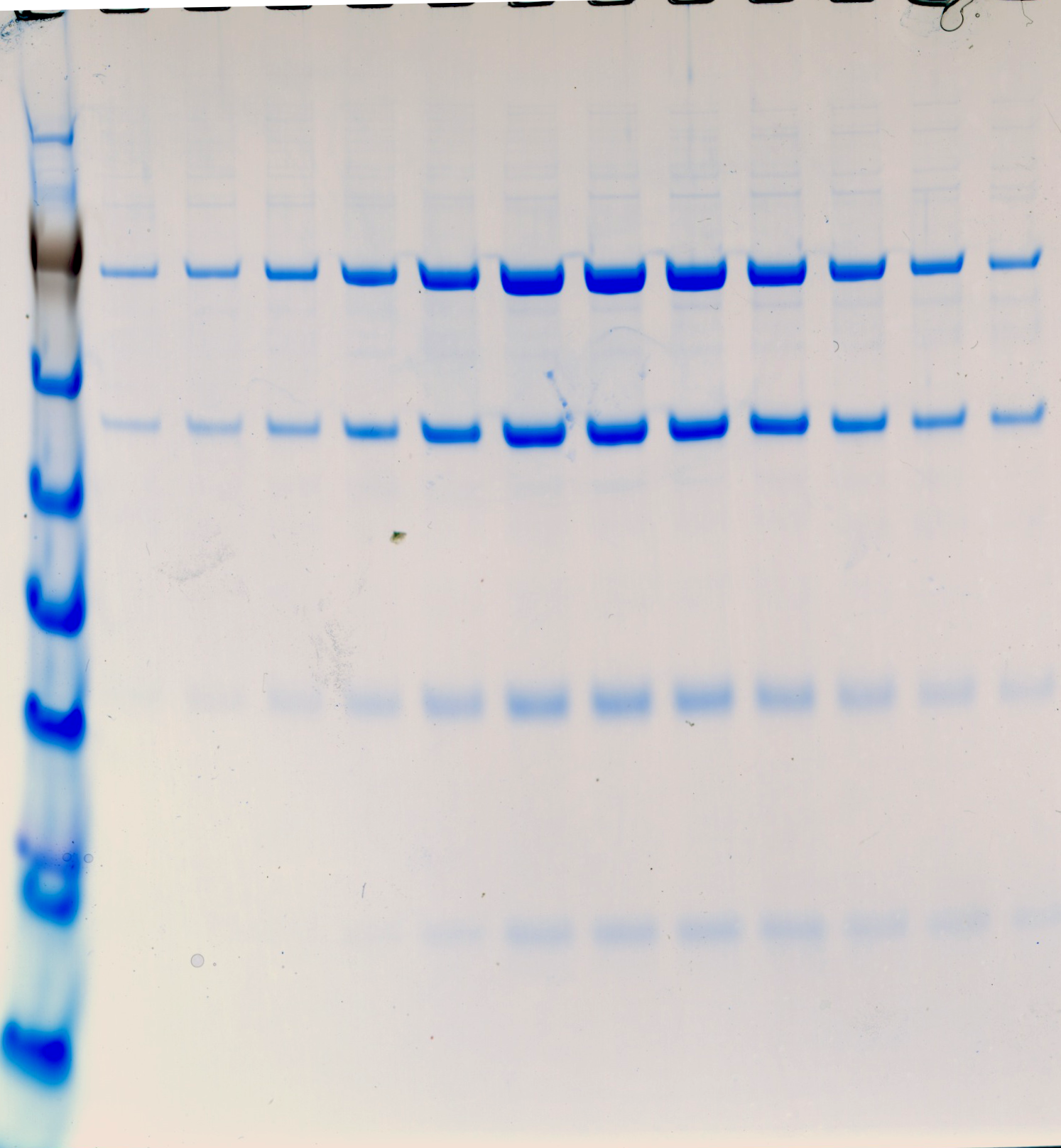

Supplement: Figure 1—source data 3. [file elife-94407-fig1-data3.zip › Figure 1 - source data 3/1C-Coomassie raw images/1C - raw images.tiff]

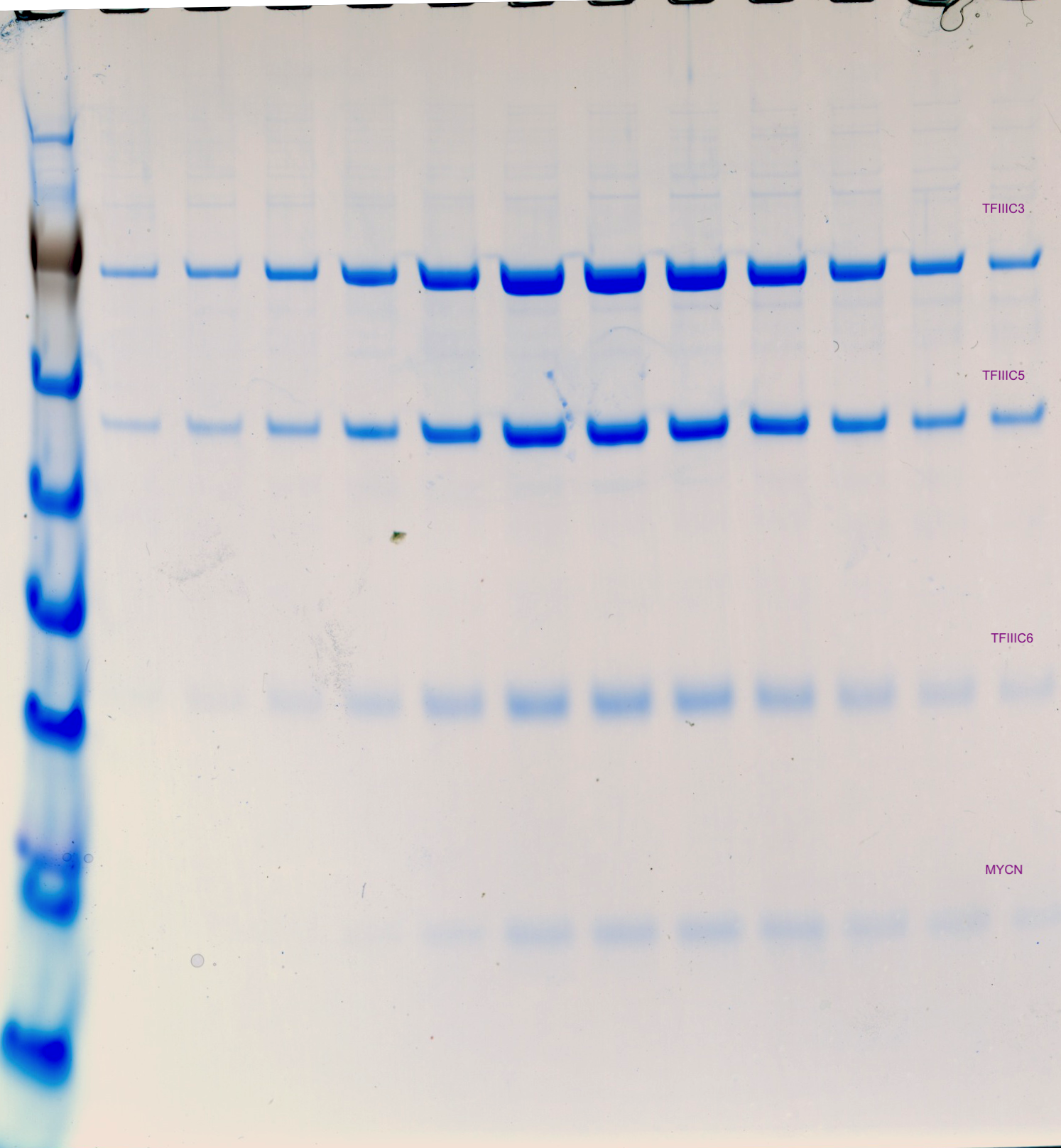

Supplement: Figure 1—source data 4. [file elife-94407-fig1-data4.zip › Figure 1 - source data 4/1C-Coomassie raw images labelled/1C - raw images labelled.tiff]

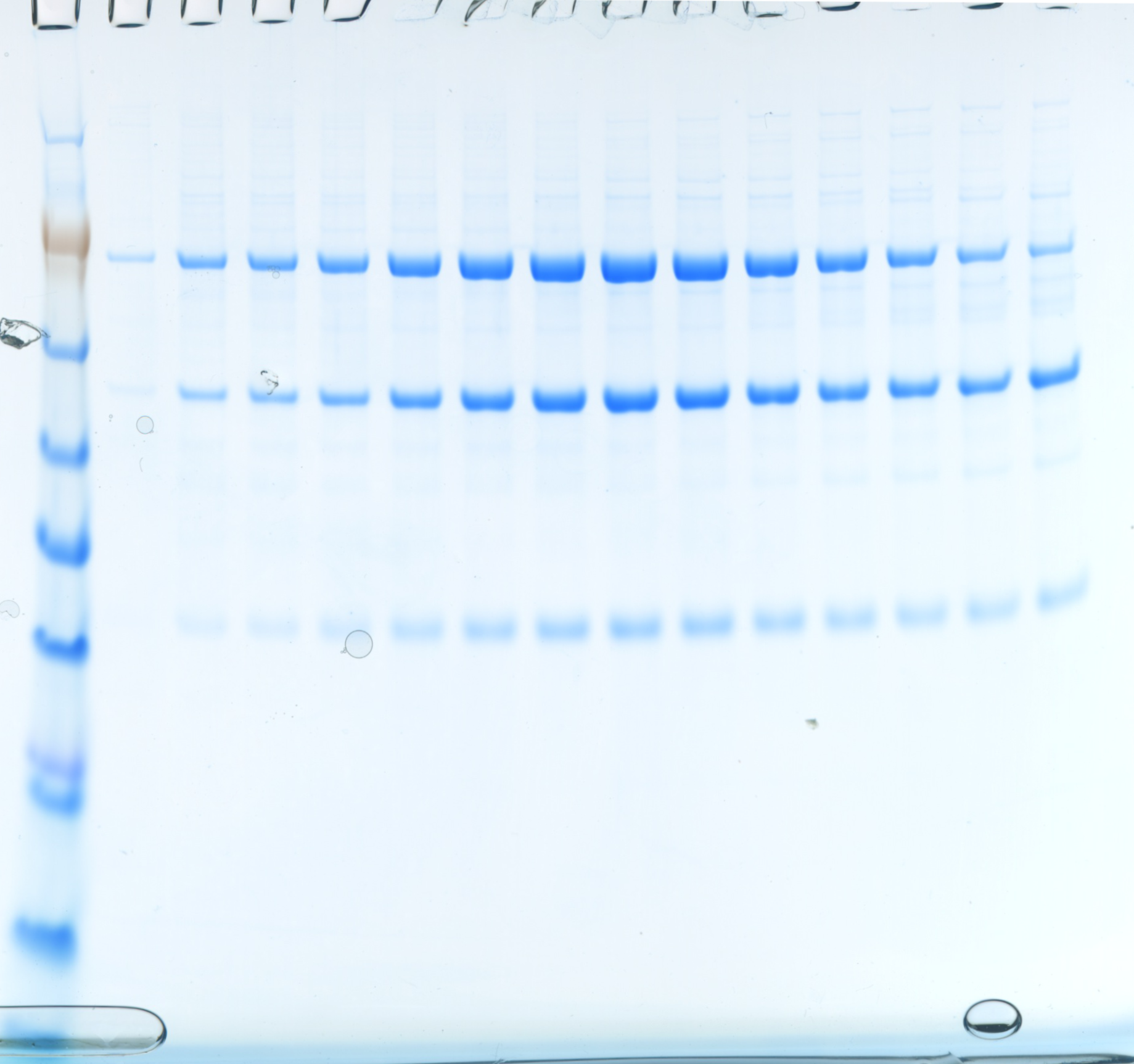

Supplement: Figure 1—figure supplement 1—source data 1. [file elife-94407-fig1-figsupp1-data1.zip › Figure 1 - supplement 1 - source data 1/S1B-Commassie raw images/FigS1B-Commassie.tiff]

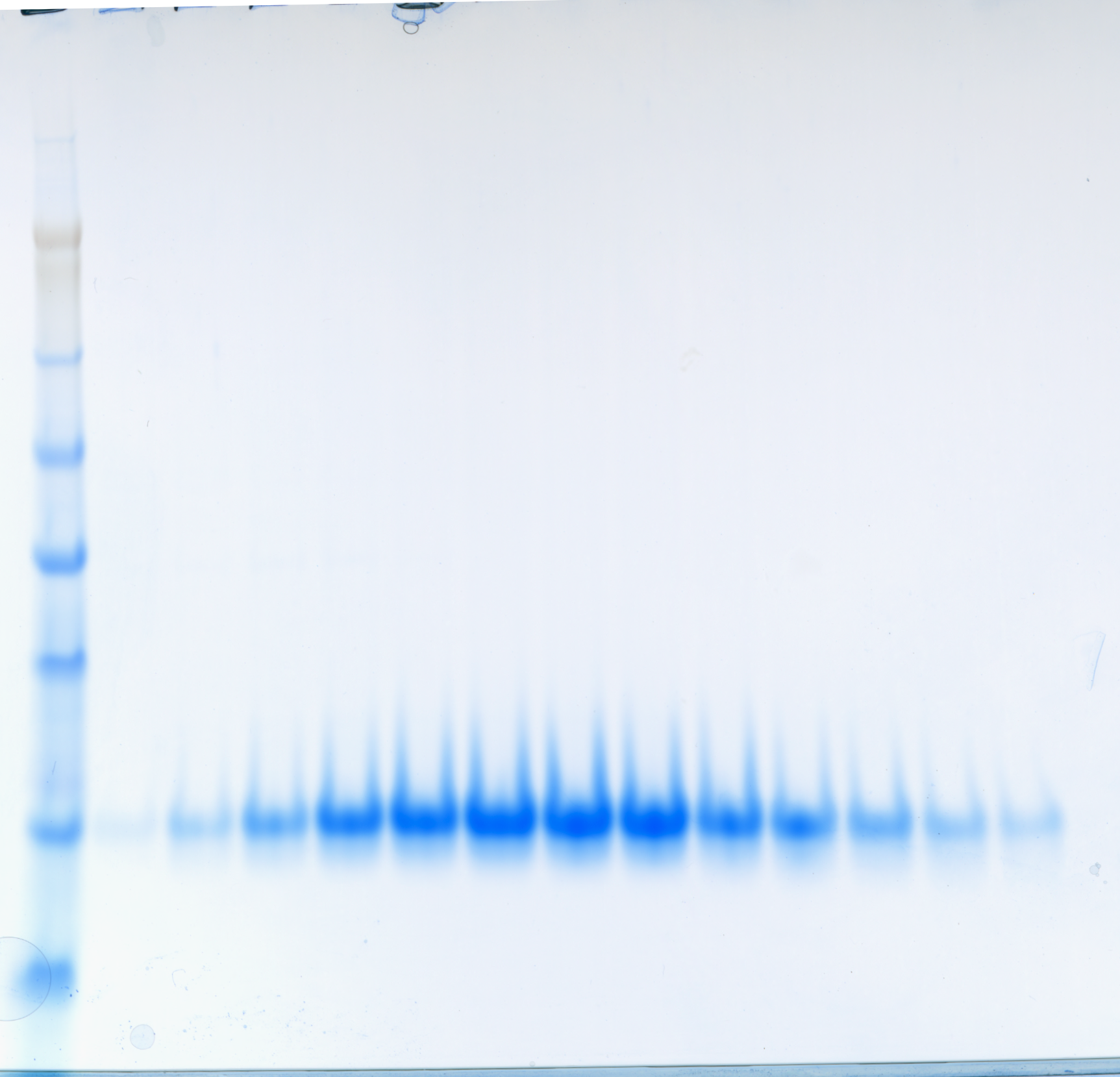

Supplement: Figure 1—figure supplement 1—source data 1. [file elife-94407-fig1-figsupp1-data1.zip › Figure 1 - supplement 1 - source data 1/S1A-Commassie raw images/Fig-S1A.tiff]

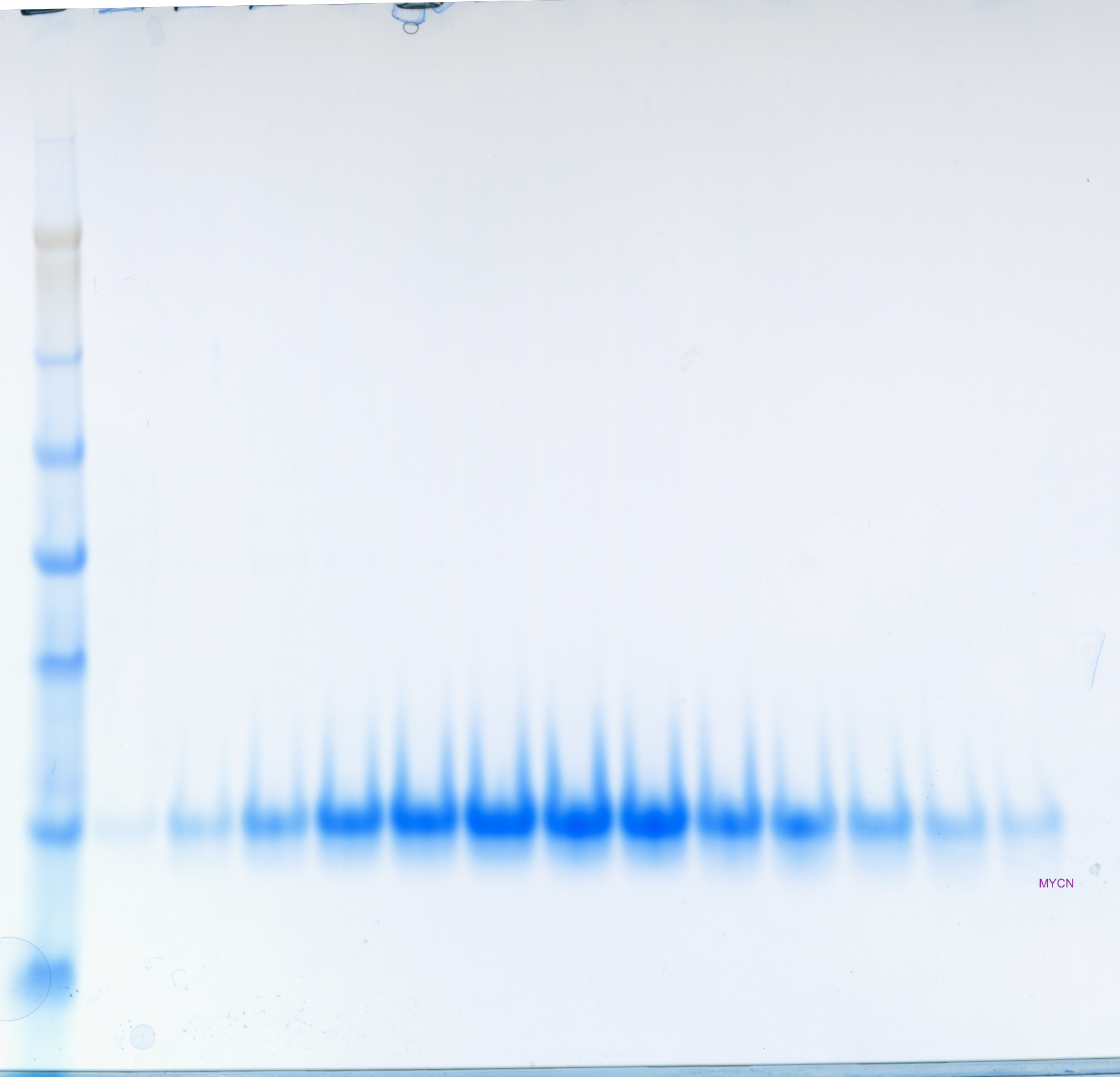

Supplement: Figure 1—figure supplement 1—source data 2. [file elife-94407-fig1-figsupp1-data2.zip › Figure 1 - supplement 1 - source data 2/S1A-Commassie raw images labelled/Fig-S1A.tiff]

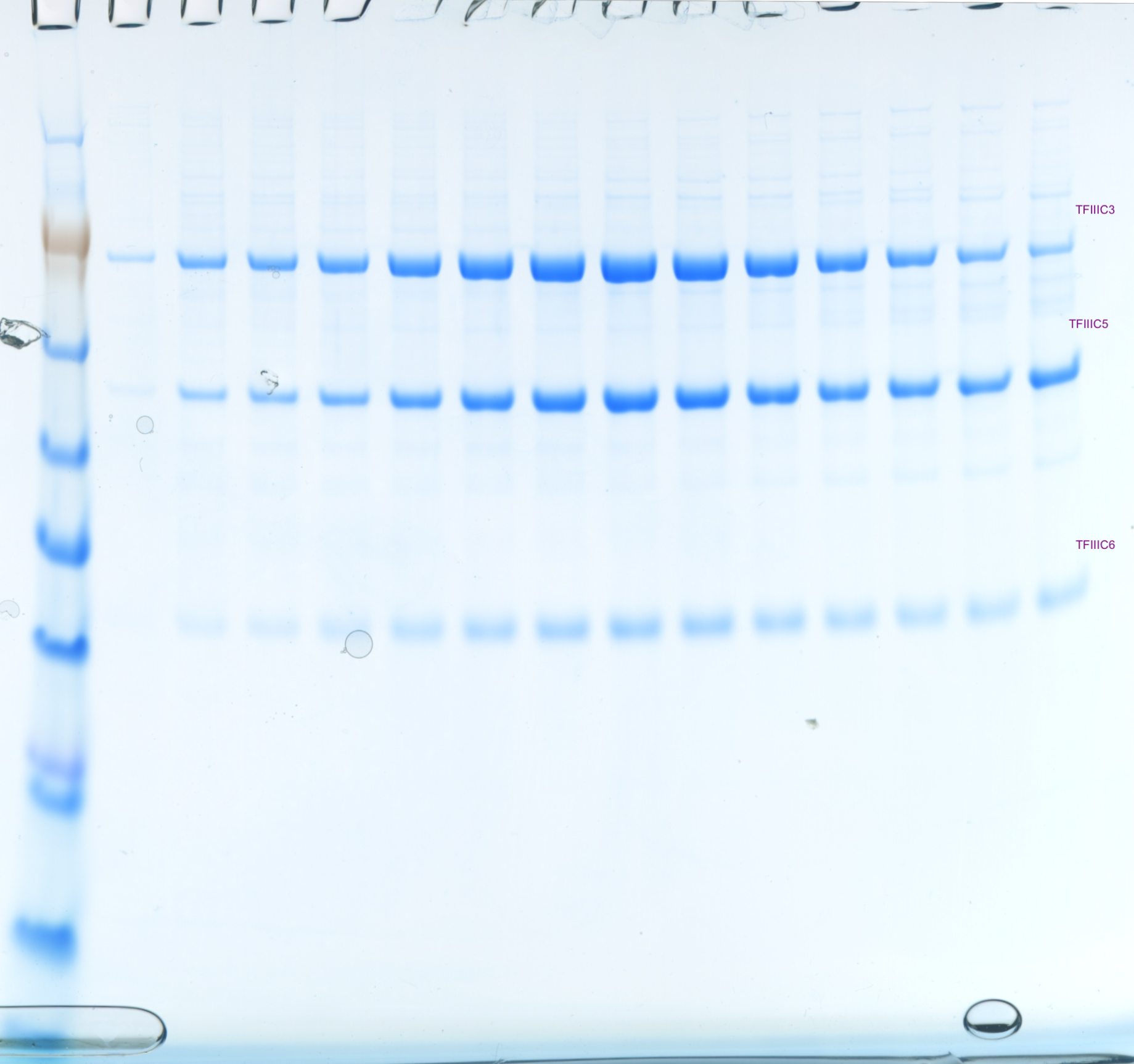

Supplement: Figure 1—figure supplement 1—source data 2. [file elife-94407-fig1-figsupp1-data2.zip › Figure 1 - supplement 1 - source data 2/S1B-Commassie raw images labelled/FigS1B-Commassie.tiff]

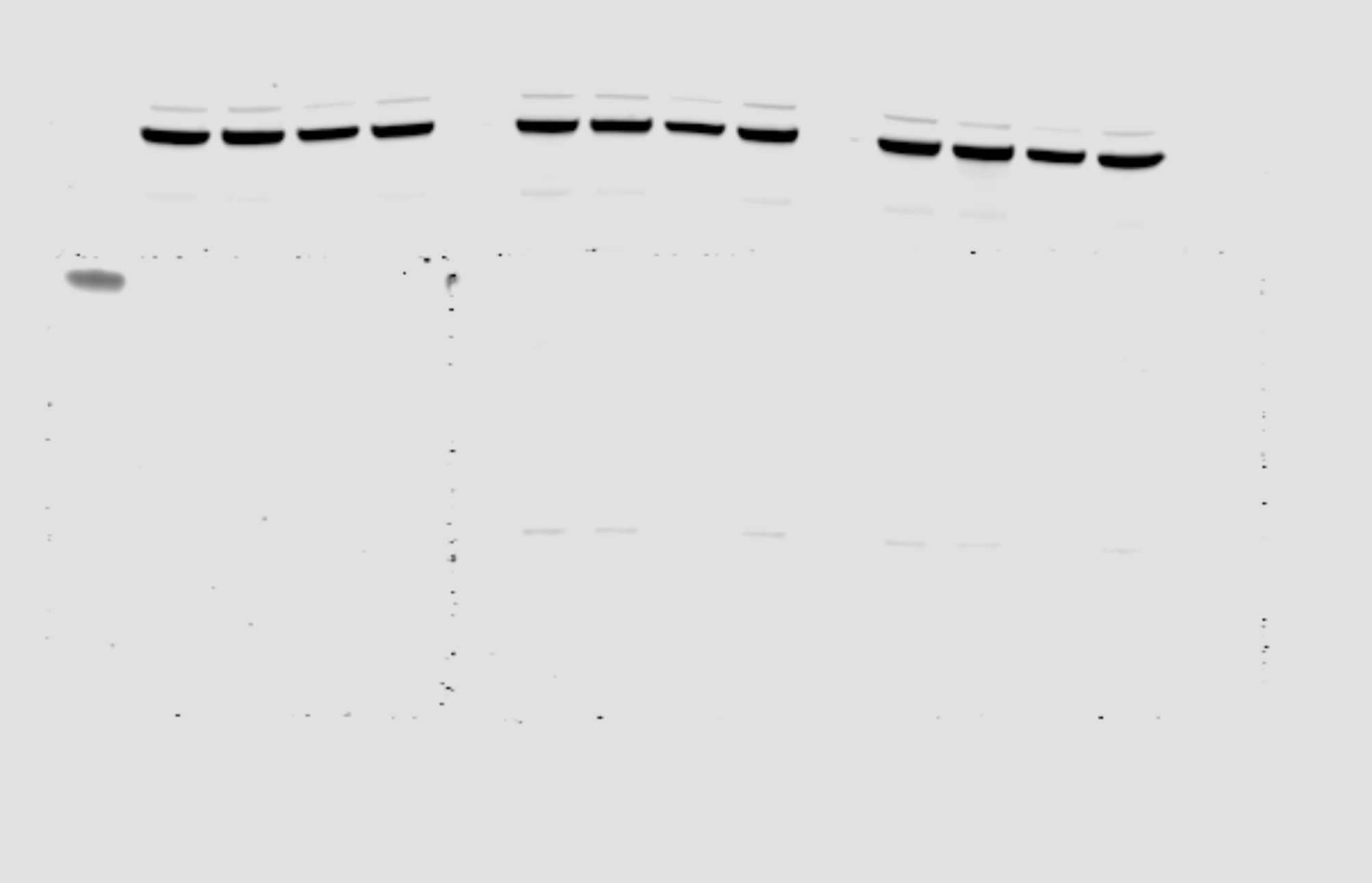

Supplement: Figure 1—figure supplement 1—source data 4. [file elife-94407-fig1-figsupp1-data4.zip › Figure 1 - supplement 1 - source data 4/S1F raw data/VCL.tif]

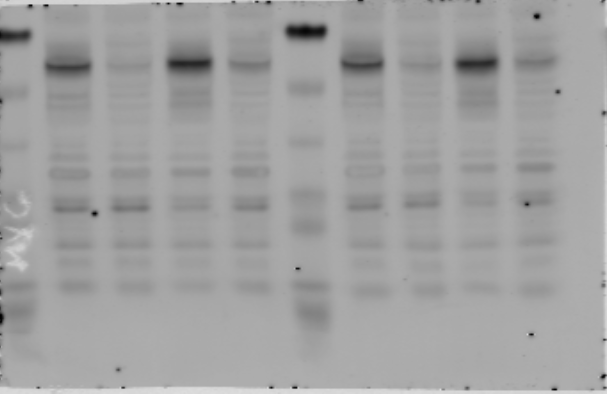

Supplement: Figure 1—figure supplement 1—source data 4. [file elife-94407-fig1-figsupp1-data4.zip › Figure 1 - supplement 1 - source data 4/S1F raw data/MYC.tif]

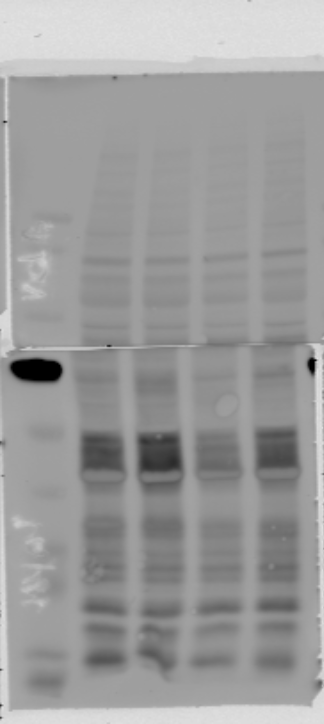

Supplement: Figure 1—figure supplement 1—source data 4. [file elife-94407-fig1-figsupp1-data4.zip › Figure 1 - supplement 1 - source data 4/S1F raw data/MYCN.tif]

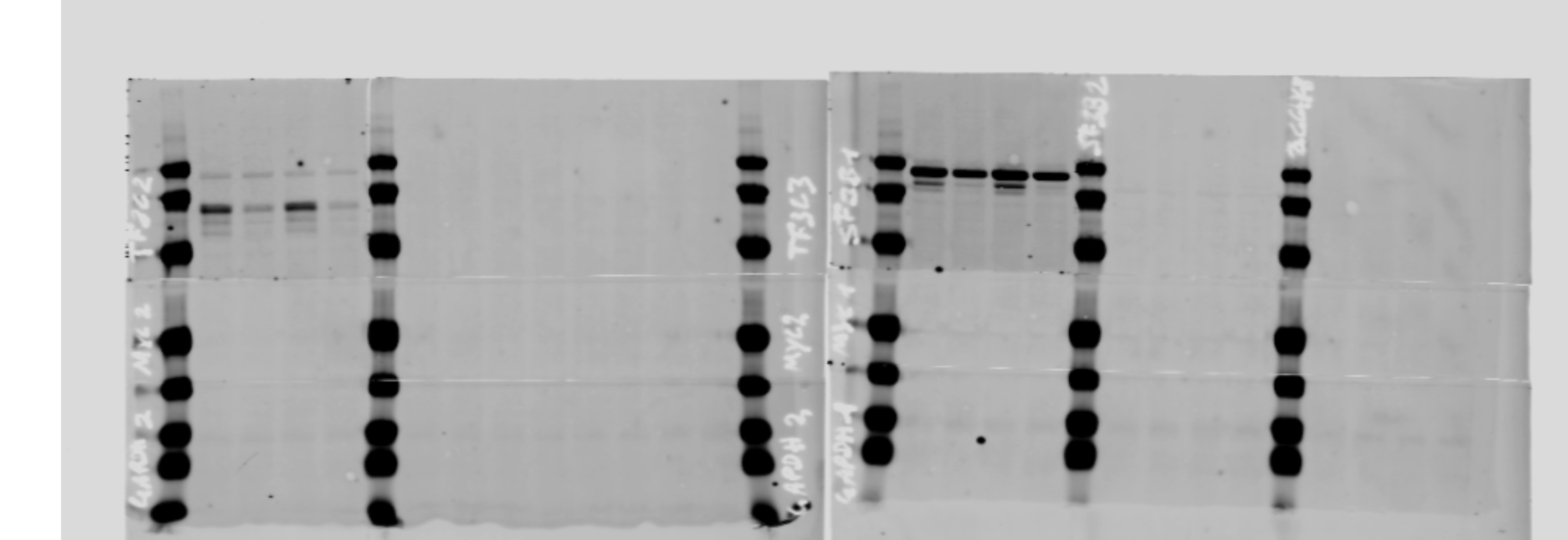

Supplement: Figure 1—figure supplement 1—source data 4. [file elife-94407-fig1-figsupp1-data4.zip › Figure 1 - supplement 1 - source data 4/S1E raw images/S1E TFIIIC2 raw images/TF3C2_I.jpg]

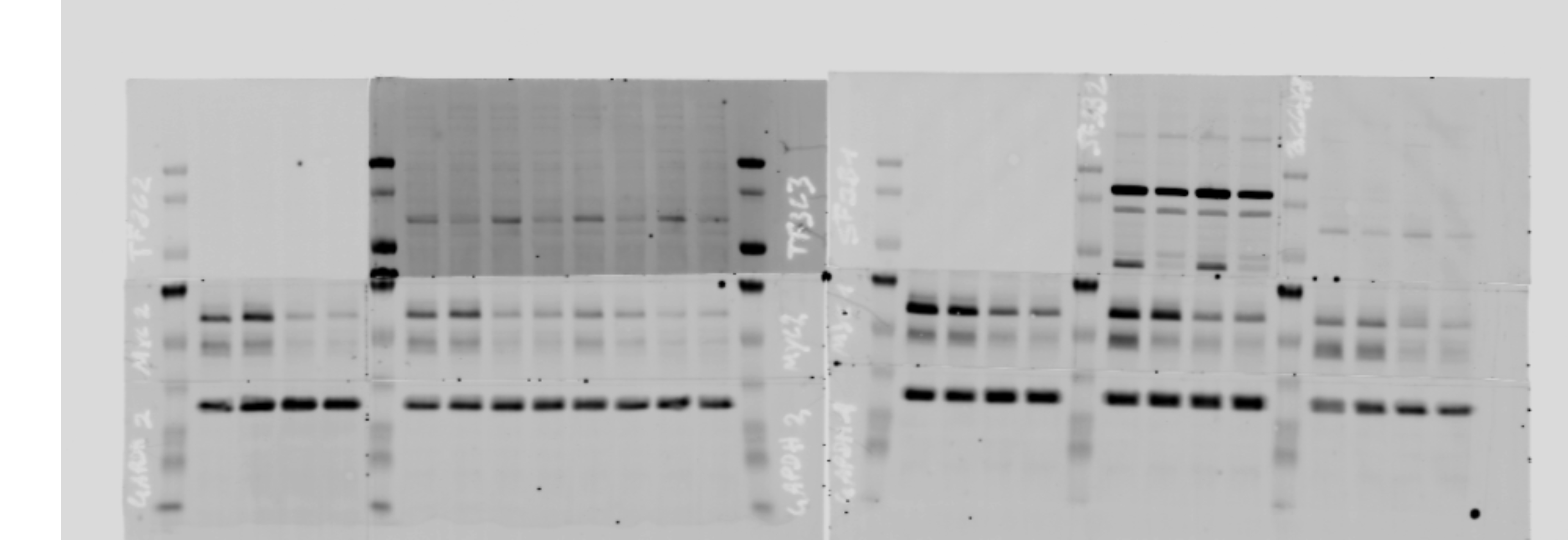

Supplement: Figure 1—figure supplement 1—source data 4. [file elife-94407-fig1-figsupp1-data4.zip › Figure 1 - supplement 1 - source data 4/S1E raw images/S1E TFIIIC2 raw images/TF3C2_III.jpg]

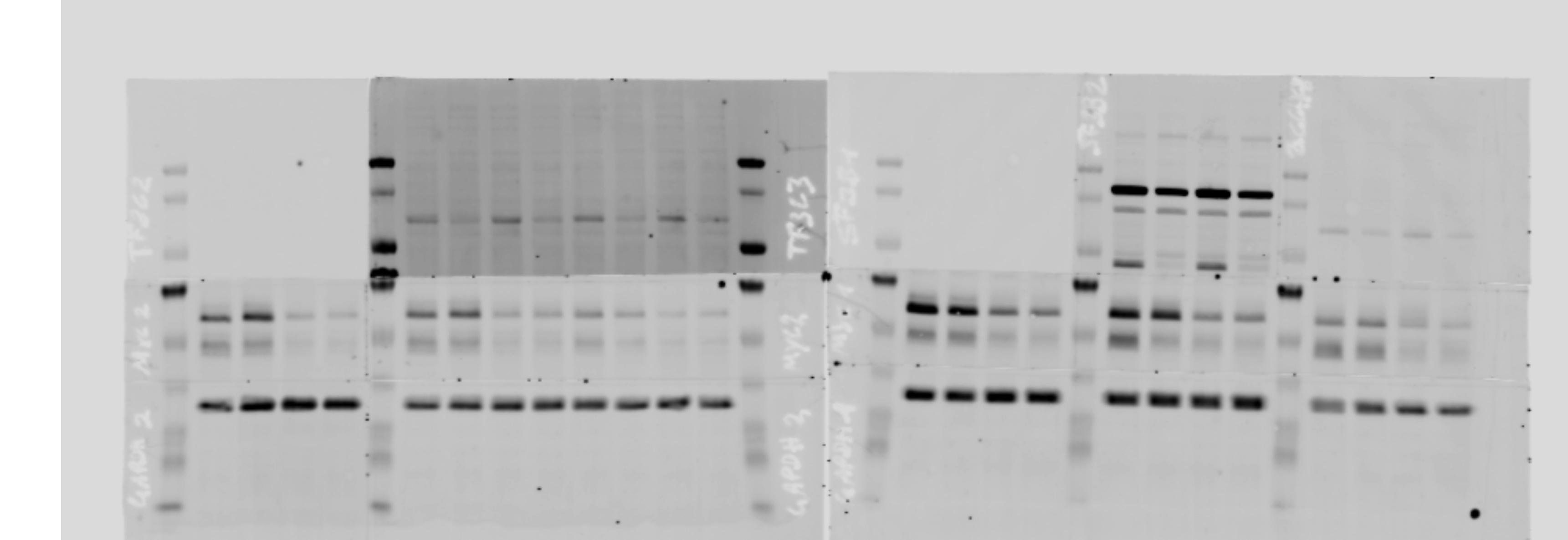

Supplement: Figure 1—figure supplement 1—source data 4. [file elife-94407-fig1-figsupp1-data4.zip › Figure 1 - supplement 1 - source data 4/S1E raw images/S1E TFIIIC2 raw images/TF3C2_II.jpg]

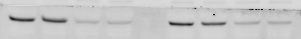

Supplement: Figure 1—figure supplement 1—source data 4. [file elife-94407-fig1-figsupp1-data4.zip › Figure 1 - supplement 1 - source data 4/S1E raw images/S1E TFIIIC5 raw images/TF3C5.png]

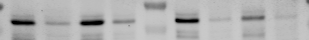

Supplement: Figure 1—figure supplement 1—source data 4. [file elife-94407-fig1-figsupp1-data4.zip › Figure 1 - supplement 1 - source data 4/S1E raw images/S1E TFIIIC5 raw images/MYC.png]

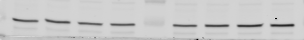

Supplement: Figure 1—figure supplement 1—source data 4. [file elife-94407-fig1-figsupp1-data4.zip › Figure 1 - supplement 1 - source data 4/S1E raw images/S1E TFIIIC5 raw images/VCL.png]

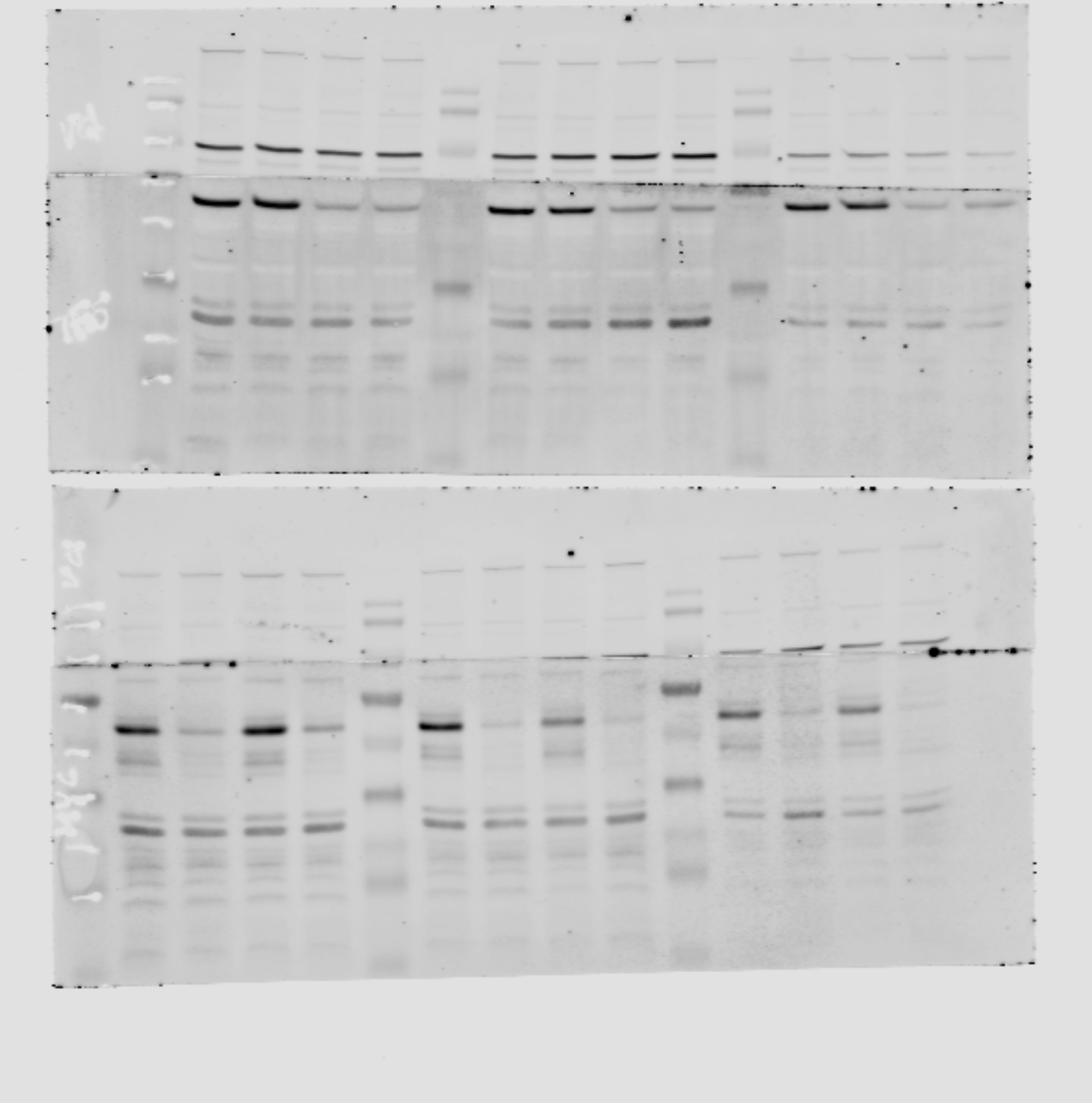

Supplement: Figure 1—figure supplement 1—source data 4. [file elife-94407-fig1-figsupp1-data4.zip › Figure 1 - supplement 1 - source data 4/S1E raw images/S1E TFIIIC5 raw images/S3E_TF3C5.tif]

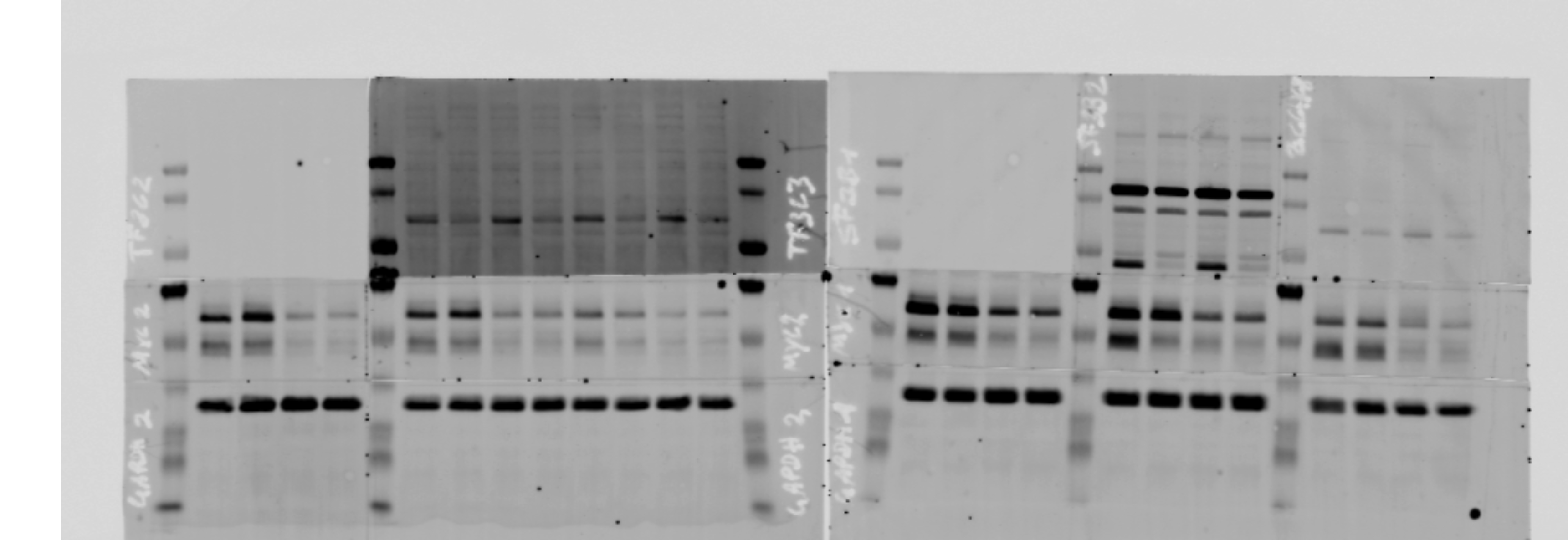

Supplement: Figure 1—figure supplement 1—source data 4. [file elife-94407-fig1-figsupp1-data4.zip › Figure 1 - supplement 1 - source data 4/S1E raw images/S1E TFIIIC3 raw images/TF3C3_I.jpg]

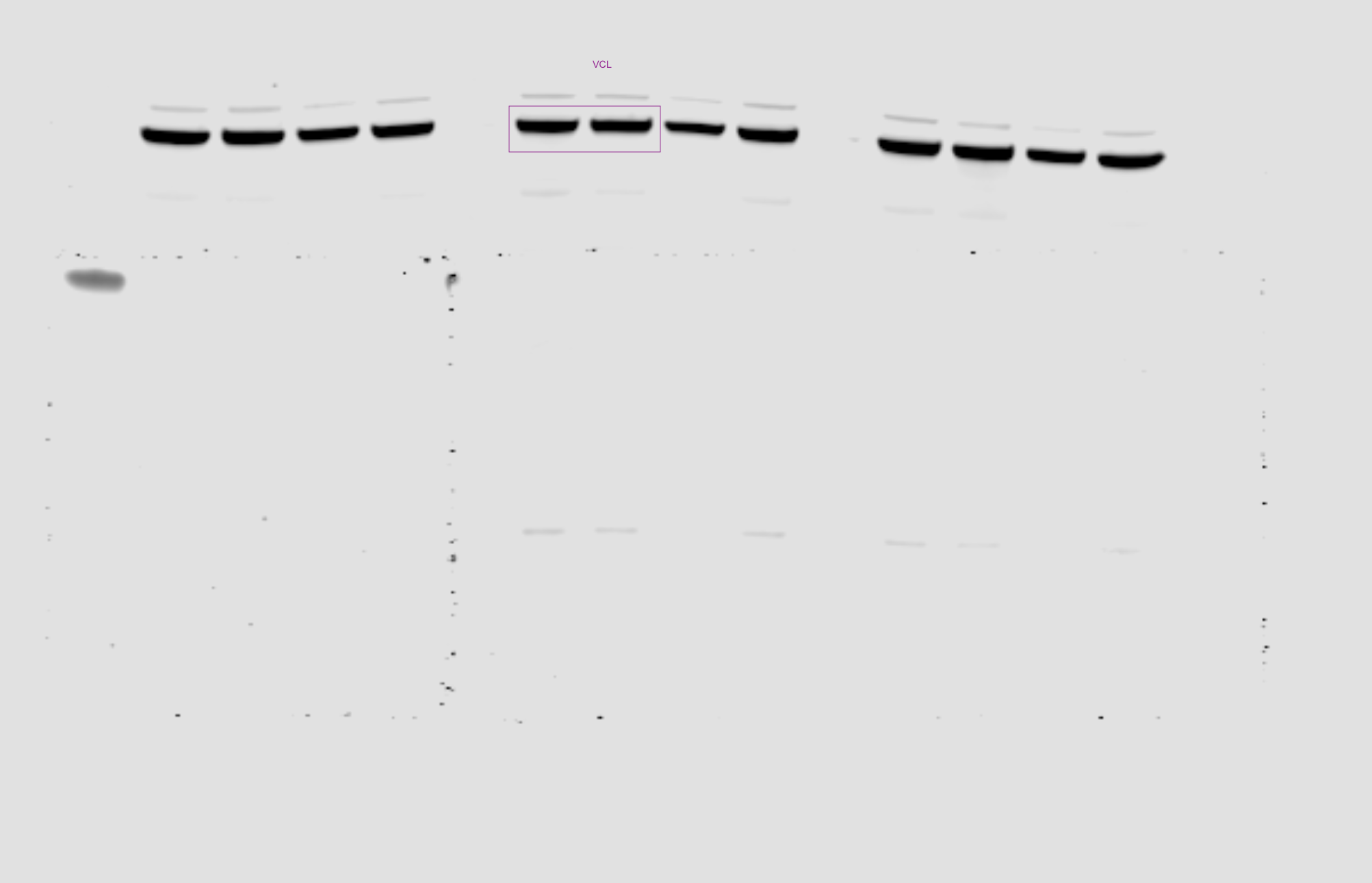

Supplement: Figure 1—figure supplement 1—source data 5. [file elife-94407-fig1-figsupp1-data5.zip › Figure 1 - supplement 1 - source data 5 /S1F raw data labelled/VCL.tif]

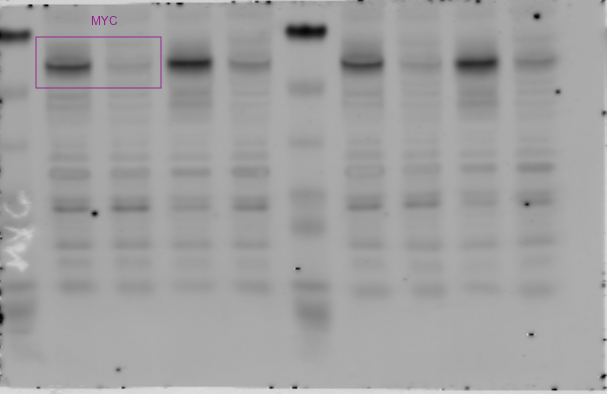

Supplement: Figure 1—figure supplement 1—source data 5. [file elife-94407-fig1-figsupp1-data5.zip › Figure 1 - supplement 1 - source data 5 /S1F raw data labelled/MYC.tif]

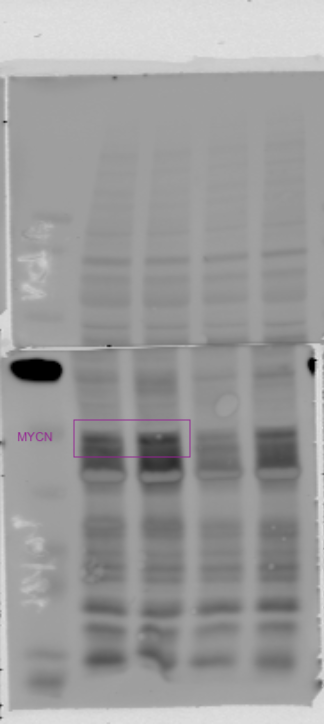

Supplement: Figure 1—figure supplement 1—source data 5. [file elife-94407-fig1-figsupp1-data5.zip › Figure 1 - supplement 1 - source data 5 /S1F raw data labelled/MYCN.tif]

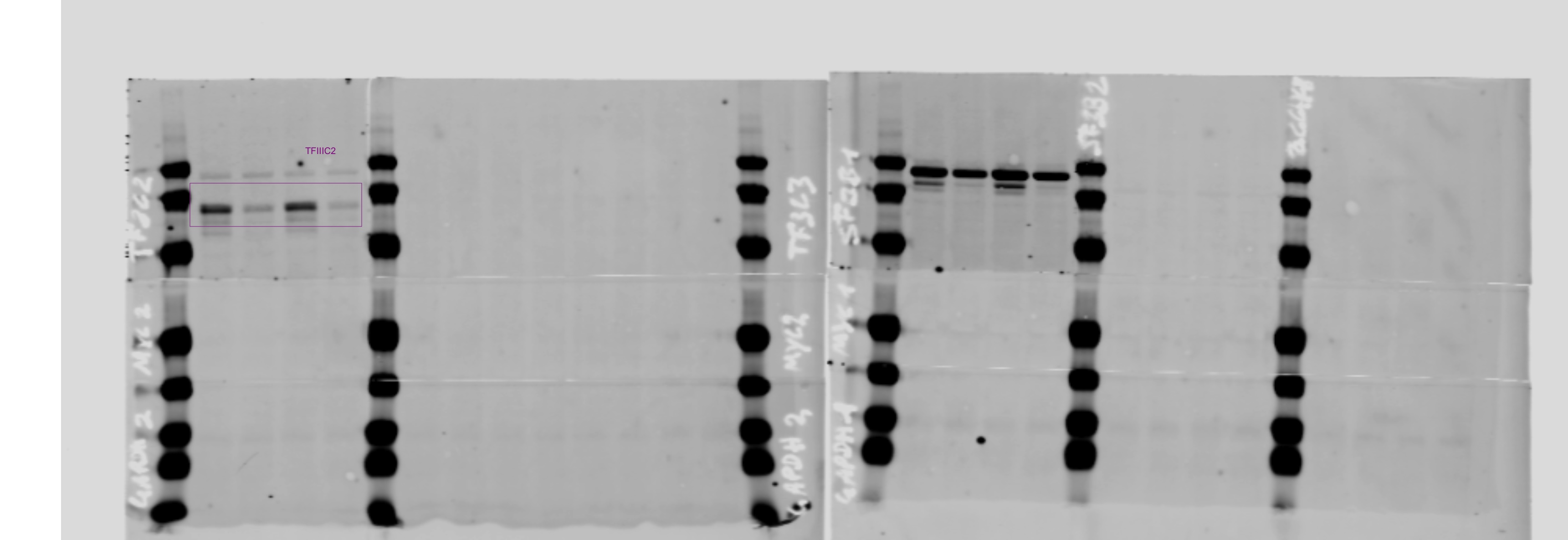

Supplement: Figure 1—figure supplement 1—source data 5. [file elife-94407-fig1-figsupp1-data5.zip › Figure 1 - supplement 1 - source data 5 /S1E raw images labelled/S1E TFIIIC2 raw images labelled/TF3C2_I.jpg]

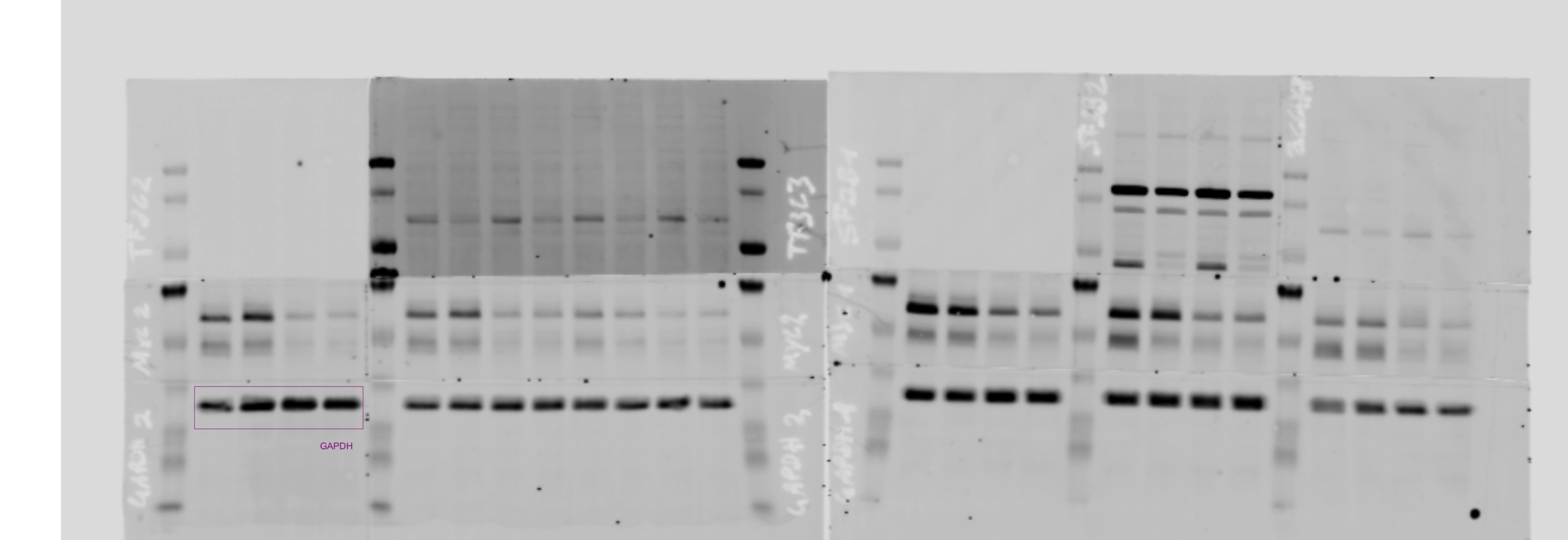

Supplement: Figure 1—figure supplement 1—source data 5. [file elife-94407-fig1-figsupp1-data5.zip › Figure 1 - supplement 1 - source data 5 /S1E raw images labelled/S1E TFIIIC2 raw images labelled/TF3C2_III.jpg]

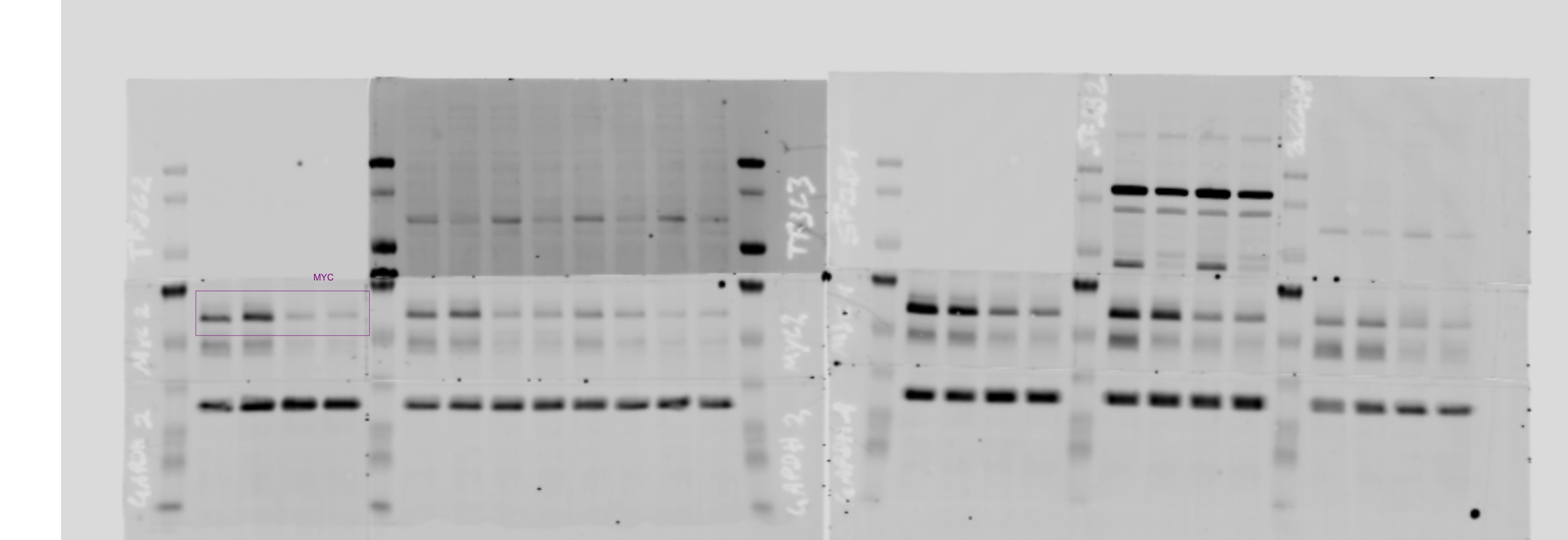

Supplement: Figure 1—figure supplement 1—source data 5. [file elife-94407-fig1-figsupp1-data5.zip › Figure 1 - supplement 1 - source data 5 /S1E raw images labelled/S1E TFIIIC2 raw images labelled/TF3C2_II.jpg]

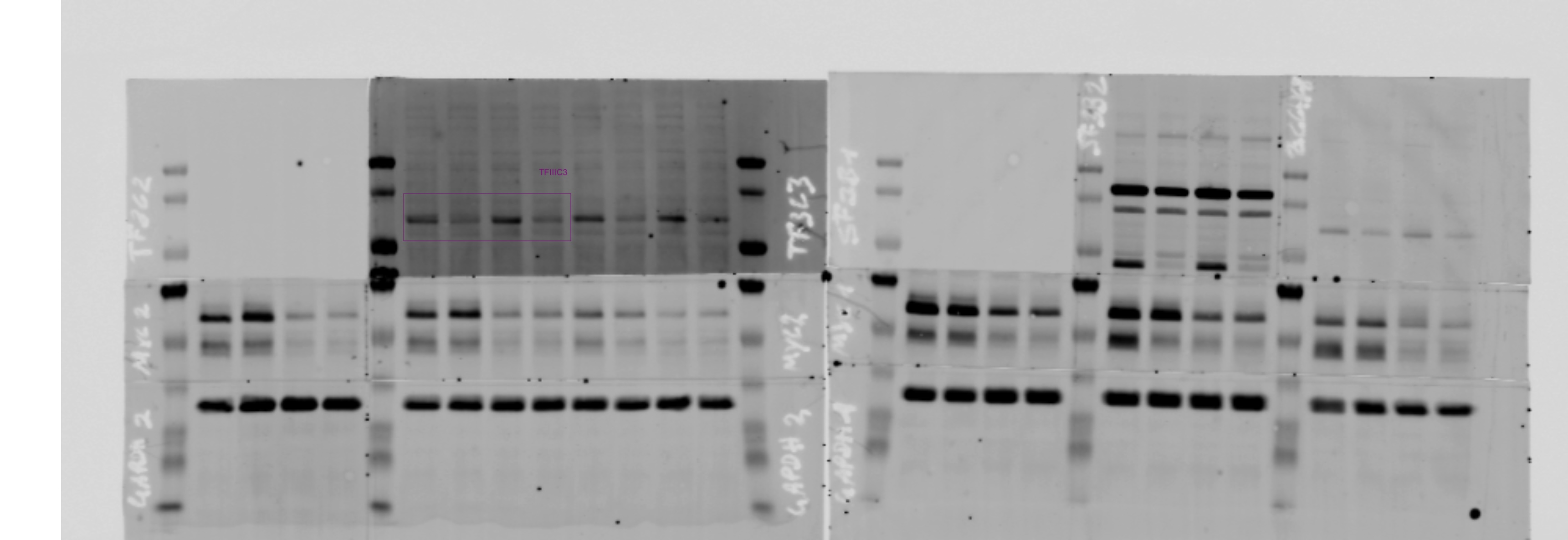

Supplement: Figure 1—figure supplement 1—source data 5. [file elife-94407-fig1-figsupp1-data5.zip › Figure 1 - supplement 1 - source data 5 /S1E raw images labelled/S1E TFIIIC3 raw images labelled/TF3C3_I.jpg]

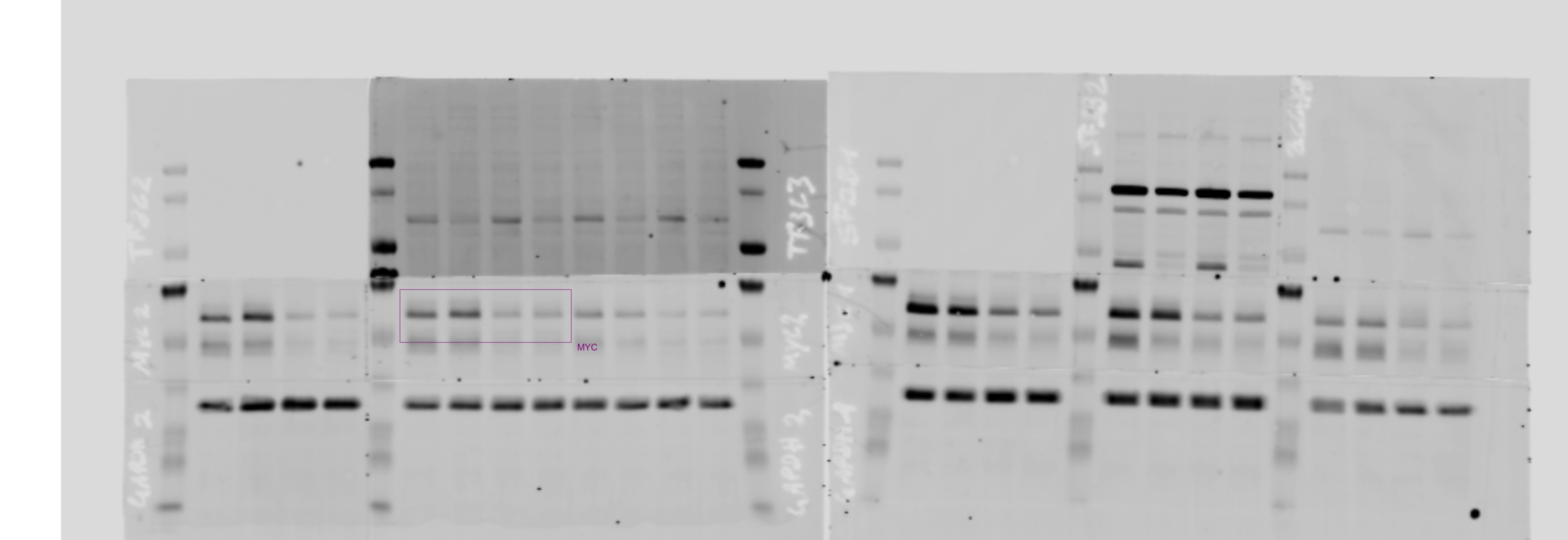

Supplement: Figure 1—figure supplement 1—source data 5. [file elife-94407-fig1-figsupp1-data5.zip › Figure 1 - supplement 1 - source data 5 /S1E raw images labelled/S1E TFIIIC3 raw images labelled/TF3C3_II.jpg]

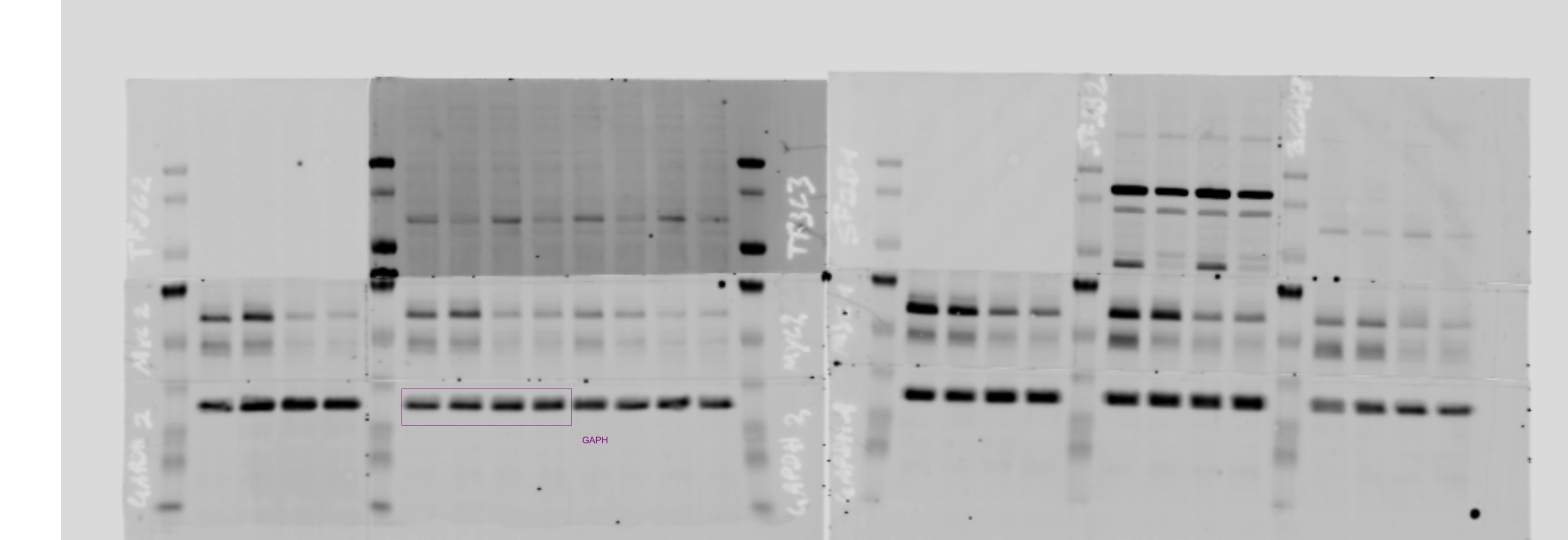

Supplement: Figure 1—figure supplement 1—source data 5. [file elife-94407-fig1-figsupp1-data5.zip › Figure 1 - supplement 1 - source data 5 /S1E raw images labelled/S1E TFIIIC3 raw images labelled/TF3C3_III.jpg]

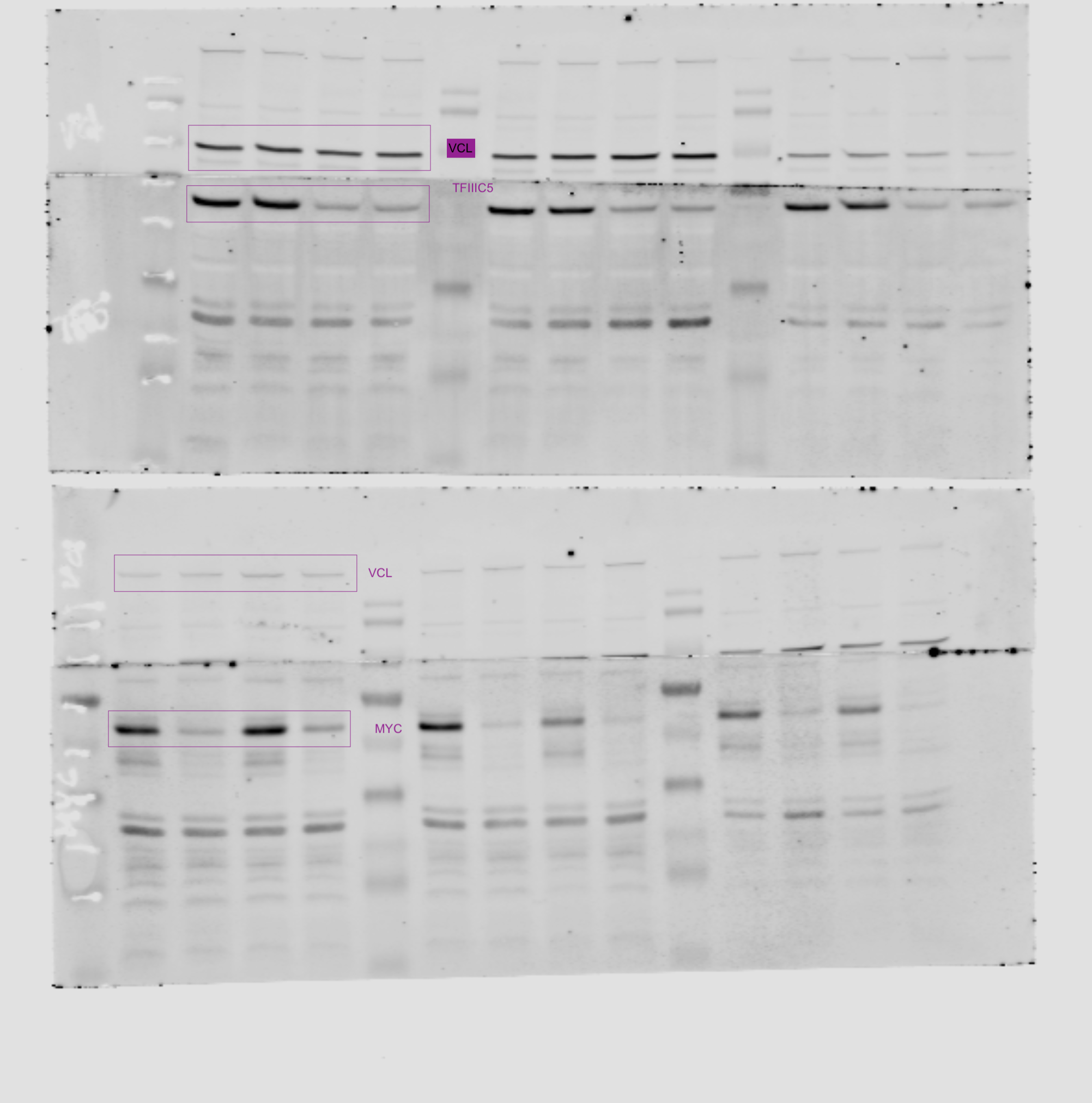

Supplement: Figure 1—figure supplement 1—source data 5. [file elife-94407-fig1-figsupp1-data5.zip › Figure 1 - supplement 1 - source data 5 /S1E raw images labelled/S1E TFIIIC5 raw images labelled/S3E_TF3C5.tif]
