# Supplementary material for: Association with TFIIIC limits MYCN localisation in hubs of active promoters and chromatin accumulation of non-phosphorylated RNA polymerase II
Source: eLife. 2024 Aug 23;13:RP94407. doi: 10.7554/eLife.94407 (PMC11343564; doi:10.7554/eLife.94407)
Supplement: Source code 1. [file elife-94407-code1.zip › Source code file 1.docx]

**Source code – Analysis of ChIP-Seq data**

#!/bin/bash

VER_BOWTIE2="pgBowtie2.sh_1.3.0"

#########################################

########## Description ##########

# functionality:

# reads list of fastq-files

# maps each fastq-file to the indicated genome, using Bowtie2

# writes bam-file as output

# sorts the output bam-file

# also writes a combined sorted bam-file, containing mapped & unmapped reads

#

# use:

# pgBowtie2.sh arg1 arg2 &>log.txt &; disown -h %1

#

# command line arguments:

# argument 1: one item per line

# row 1: full path to fastq directory

# row 2: full path to output-directory (bam- & sam-directory)

# row 3: full path to user directory; optional - if no directory is provided, the "output directory" will be used

# row 4: full path to bowtie2 genome

# row 5: additional tophat/bowtie2 parameters (optional)

# row 6: empty (\n)

# argument 2: list of fastq-files

# row 1 ff: one fastq-file name per row (root name, without extension '.fastq')

# last row: empty (\n)

# retrieve parameters

fastq_dir=`sed -n '1p' < $1`

bam_dir=`sed -n '2p' < $1`

user_dir=`sed -n '3p' < $1`

genome=`sed -n '4p' < $1`

parameters=`sed -n '5p' < $1`

samples=$2

#map with tophat1/bowtie2

while read name; do

if [ -f "${fastq_dir}/${name}.fastq" ]; then

printf "run tophat with the parameters: --no-coverage-search -p 40 $parameters for sample $name...\n"

tophat --no-coverage-search -g 1 -p 40 -o ${bam_dir}/${name} $genome ${fastq_dir}/${name}.fastq 2>${bam_dir}/${name}_tophat_report.txt

fi

done < $samples

#sort and index bam file

printf "create sorted .bam files...\n"

while read name; do

if [ -f "${bam_dir}/${name}/accepted_hits.bam" ]; then

samtools sort -@ 35 ${bam_dir}/${name}/accepted_hits.bam -o ${bam_dir}/${name}/${name}.mapped_sort.bam

samtools index ${bam_dir}/${name}/${name}.mapped_sort.bam

samtools sort -@ 35 ${bam_dir}/${name}/unmapped.bam -o ${bam_dir}/${name}/unmapped_sort.bam

samtools merge -@ 35 ${bam_dir}/${name}_sort.bam ${bam_dir}/${name}/${name}.mapped_sort.bam ${bam_dir}/${name}/unmapped_sort.bam

samtools index ${bam_dir}/${name}_sort.bam

# set extended attributes

old_attributes=`getfattr -n user.comment ${fastq_dir}/${name}.fastq | sed '1d' | sed 's/user.comment=//' | sed 's/\"//g'`

date=`date +%y%m%d`

added_attributes="<MOD>$VER_BOWTIE2</MOD><DAT>$date</DAT><GEN>$genome</GEN><FIL>${fastq_dir}/${name}.fastq</FIL><PAR>$parameters</PAR>"

new_attributes="${old_attributes}${added_attributes}"

setfattr -n user.comment -v "$new_attributes" ${bam_dir}/${name}_sort.bam

setfattr -n user.comment -v "$new_attributes" ${bam_dir}/${name}_sort.bam.bai

fi

done < $samples

printf "%s\n" $VER_BOWTIE2 > $user_dir/ver_bowtie2#!/bin/bash

VER_CHIPSEQ_SPIKEIN_MOUSE="pgChIPseq_spikein_mouse.sh_1.2.0"

### NOTE: thread numbers reduced in "pgBowtie1.sh" & in lines 150, 151, 206, 207, 324, 370, 371 (03.12.2019)

########################################

########## description #########

# functionality:

# maps the fastq-files to the mm10 (bowtie1, for ungapped alignment)

# takes the unmapped reads (mm10): maps them to hg19

# takes the mapped reads (mm10): maps them to hg19

# establishes mapping stats

#

# command line arguments:

# argument 1: dir_file

# one column only

# row 1: fastq_dir = absolute path to directory with fastq-files; default is "$user_dir/O1_fastq"

# row 2: bam_dir = absolute path to directory with bam-files; default is "$user_dir/02_bam"

# row 3: mapstat_dir = absolute path to directory with mapstats-output; default is "$user_dir/05_stats"

# row 4: bg_dir = absolute path to directory with bedgraph-output; default is "$user_dir/03_bedgraph"

#

# argument 2: info_file

# row 1: bedgraph_flag: if set to "G": bedgraph will be established

# row 2: experiment: name to be used for mapstats output

#

# argument 3: sample_file

# col1: sample = root part of starting data file;

# - the part before the extension ".fastq"

#

# scripts used:

# pgBowtie1.sh

# pgMapStats.sh

##################################################################

########## define common data & read input data ##########

# dir_file

fastq_dir=`sed -n '1p'< $1`

bam_dir=`sed -n '2p'< $1`

mapstat_dir=`sed -n '3p'< $1`

bg_dir=`sed -n '4p'< $1`

bedgraph_flag=`sed -n '1p'< $2`

experiment=`sed -n '2p'< $2`

# sample_file

sample_file=$3

processed_sample_file=$sample_file

# define some commen variables & paths

bowtie1_dir="/media/user/data/_genomes/bowtie1"

##############################################

########## start of the program ##########

start=`date +%s`

logname=`date +%y%m%d_%H%M%S`

user_dir=`pwd`

log_long="${user_dir}/log_pgChIPseq_spikein_mouse_${logname}_ERROR.txt"

exec &>$log_long

printf "%s\n%s %s\n\n" "start:" `date +%d/%m/%Y,%H:%M:%S` >> $log_long

log_short="${user_dir}/log_pgChIPseq_spikein_mouse_${logname}_short.txt"

printf "%s\n%s %s\n" $VER_CHIPSEQ_SPIKEIN_MOUSE "start:" `date +%d/%m/%Y,%H:%M:%S` > $log_short

contents_dir_file=`cat $1`

contents_info_file=`cat $2`

contents_sample_file=`cat $3`

printf "%s\n%s\n\n%s\n%s\n%s\n%s\n\n%s\n\n" "dir_file:" "$contents_dir_file" "info_file:" "$contents_info_file" "sample_file:" "$contents_sample_file" >> $log_short

############################################################

########## 1st call of pgBowtie1.sh: mm10 ##########

#

# command line arguments:

# argument 1: one item per line

# row 1: full path to fastq directory

# row 2: full path to output-directory (bam- & sam-directory)

# row 3: full path to user directory; optional - if no directory is provided, the "output directory" will be used

# row 4: full path to bowtie1 genome

# row 5: additional tophat/bowtie2 parameters (optional)

# row 6: empty (\n)

# argument 2: list of fastq-files

# row 1 ff: one fastq-file name per row (root name, without extension '.fastq')

# last row: empty (\n)

# define the command line variables

if [ ! -d "${bam_dir}/mm10" ]; then

mkdir -p ${bam_dir}/mm10

fi

printf "%s\n%s\n%s\n%s\n" "$fastq_dir" "$bam_dir/mm10" "$user_dir" "$bowtie1_dir/mm10" > ${user_dir}/arg11_bowtie1

awk '{OFS="\t"} {print $1}' $3 > ${user_dir}/arg12_bowtie1

# run the script

printf "%s\n\n" "pgChIPseq_spikein_mouse.sh:mm10" >> $log_long

pgBowtie1.sh ${user_dir}/arg11_bowtie1 ${user_dir}/arg12_bowtie1

printf "%s\n" " " >> $log_short

cat "$user_dir/ver_bowtie1" >> $log_short

cat ${user_dir}/arg1*_bowtie1 >> $log_short

rm ${user_dir}/arg1*_bowtie1

rm "$user_dir/ver_bowtie1"

##########################################################################

########## 1st call of pgMapstats.sh for 'mapped to mm10' ##########

#

# command line arguments:

# argument 1: one item per line

# row 1: full path to input directory (containing sorted bam-files with index)

# row 2: full path to output directory

# row 3: experiment name - to be used as the file name for output

# row 4: full path to user directory

# argument 2: sample_file

# row 1 ff: full name of bam-file (including extension ".bam")

# last row: empty (\n)

if [ ! -d "$mapstat_dir" ]; then

mkdir -p $mapstat_dir # create the output-directory if it doesn't exist yet

fi

printf "%s\n%s\n%s\n%s\n" "${bam_dir}/mm10" "$mapstat_dir" "${experiment}_mapped_mm10" "$user_dir" > ${user_dir}/arg11_mapstats

awk '{OFS="\t"} {print $1"_sort.bam"}' $3 > ${user_dir}/arg12_mapstats

# run the script

printf "%s\n\n" "pgMapstats.sh mapped_mm10" >> $log_long

pgMapstats.sh arg11_mapstats arg12_mapstats &>>$log_long

printf "%s\n\n" " " >> $log_short

cat "$user_dir/ver_mapstats" >> $log_short

cat ${user_dir}/arg1*_mapstats >> $log_short

rm ${user_dir}/arg1*_mapstats

rm "$user_dir/ver_mapstats"

min=`cat "${mapstat_dir}/${experiment}_mapped_mm10.min_mapped_reads.txt"`

printf "%s\t%s\n" "min_mapped_reads $genome:" $min >> $log_short

####################################################################

##### establish fastq-files: mapped_mm10 & unmapped_mm10 #####

#

if [ ! -d "${bam_dir}/mm10/unmapped_mm10" ]; then

mkdir -p ${bam_dir}/mm10/unmapped_mm10

fi

if [ ! -d "${bam_dir}/mm10/mapped_mm10" ]; then

mkdir -p ${bam_dir}/mm10/mapped_mm10

fi

while read sample; do

samtools bam2fq -@ 5 -f 4 $bam_dir/mm10/${sample}_sort.bam > ${bam_dir}/mm10/unmapped_mm10/${sample}_unmapped_mm10.fastq # original: -@ 20

samtools bam2fq -@ 5 -F 4 $bam_dir/mm10/${sample}_sort.bam > ${bam_dir}/mm10/mapped_mm10/${sample}_mapped_mm10.fastq # original: -@ 20

# add extended attributes

old_attributes=`getfattr -n user.comment $bam_dir/mm10/${sample}_sort.bam | sed '1d' | sed 's/user.comment=//' | sed 's/\"//g'`

date=`date +%y%m%d`

added_attributes1="<MOD>$VER_CHIPSEQ_SPIKEIN_MOUSE</MOD><DAT>$date</DAT><GEN>unmapped_to_mm10</GEN><FIL>$bam_dir/mm10/${sample}_sort.bam</FIL>"

added_attributes2="<MOD>$VER_CHIPSEQ_SPIKEIN_MOUSE</MOD><DAT>$date</DAT><GEN>mapped_to_mm10</GEN><FIL>$bam_dir/mm10/${sample}_sort.bam</FIL>"

new_attributes1="${old_attributes}${added_attributes1}"

new_attributes2="${old_attributes}${added_attributes2}"

setfattr -n user.comment -v "$new_attributes1" ${bam_dir}/mm10/unmapped_mm10/${sample}_unmapped_mm10.fastq

setfattr -n user.comment -v "$new_attributes2" ${bam_dir}/mm10/mapped_mm10/${sample}_mapped_mm10.fastq

done < $sample_file

##################################################################################

########## 2nd call of pgBowtie1.sh: unmapped_mm10 -> map to hg19 ##########

#

# command line arguments:

# argument 1: one item per line

# row 1: full path to fastq directory

# row 2: full path to output-directory (bam- & sam-directory)

# row 3: full path to user directory; optional - if no directory is provided, the "output directory" will be used

# row 4: full path to bowtie1 genome

# row 5: additional tophat/bowtie2 parameters (optional)

# argument 2: list of fastq-files

# row 1 ff: one fastq-file name per row (root name, without extension '.fastq')

# define the command line variables

if [ ! -d "${bam_dir}/hg19/unmapped_mm10" ]; then

mkdir -p ${bam_dir}/hg19/unmapped_mm10

fi

printf "%s\n%s\n%s\n%s\n" "${bam_dir}/mm10/unmapped_mm10" "${bam_dir}/hg19/unmapped_mm10" "$user_dir" "$bowtie1_dir/hg19" > ${user_dir}/arg21_bowtie1

awk '{OFS="\t"} {print $1"_unmapped_mm10"}' $3 > ${user_dir}/arg22_bowtie1

# run the script

printf "%s\n\n" "pgChIPseq_spikein_mouse.sh:hg19_unmapped_mm10" >> $log_long

pgBowtie1.sh ${user_dir}/arg21_bowtie1 ${user_dir}/arg22_bowtie1

printf "%s\n" " " >> $log_short

cat "$user_dir/ver_bowtie1" >> $log_short

cat ${user_dir}/arg2*_bowtie1 >> $log_short

rm ${user_dir}/arg2*_bowtie1

rm "$user_dir/ver_bowtie1"

###################################################################################

##### establish bam-files: mapped_hg19_only = unmapped_mm10 -> mapped_hg19 #####

#

if [ ! -d "${bam_dir}/hg19/mapped_hg19_only" ]; then

mkdir -p ${bam_dir}/hg19/mapped_hg19_only

fi

while read sample; do

samtools view -@ 5 -F 4 -O BAM -o ${bam_dir}/hg19/mapped_hg19_only/${sample}_only_hg19.bam $bam_dir/hg19/unmapped_mm10/${sample}_unmapped_mm10_sort.bam # original: -@ 20

samtools sort -@ 5 ${bam_dir}/hg19/mapped_hg19_only/${sample}_only_hg19.bam -o ${bam_dir}/hg19/mapped_hg19_only/${sample}_only_hg19_sort.bam # original: -@ 20

rm ${bam_dir}/hg19/mapped_hg19_only/${sample}_only_hg19.bam

samtools index ${bam_dir}/hg19/mapped_hg19_only/${sample}_only_hg19_sort.bam

mappedreads=`samtools idxstats ${bam_dir}/hg19/mapped_hg19_only/${sample}_only_hg19_sort.bam | grep -v "*" | awk '{s+=$3} END {print s}'`

unmappedreads=`samtools idxstats ${bam_dir}/hg19/mapped_hg19_only/${sample}_only_hg19_sort.bam | grep "*" | awk '{print $4}'`

total=`echo $((mappedreads+unmappedreads))`

# add extended attributes

old_attributes=`getfattr -n user.comment $bam_dir/hg19/mapped_mm10/${sample}_unmapped_mm10_sort.bam | sed '1d' | sed 's/user.comment=//' | sed 's/\"//g'`

date=`date +%y%m%d`

added_attributes1="<MOD>$VER_CHIPSEQ_SPIKEIN_MOUSE</MOD><GEN>only_mapped_to_hg19</GEN><FIL>$bam_dir/hg19/unmapped_mm10/${sample}_unmapped_mm10_sort.bam</FIL><NUM>$total</NUM><MAP>$mappedreads</MAP><UNM>$unmappedreads</UNM>"

new_attributes1="${old_attributes}${added_attributes1}"

setfattr -n user.comment -v "$new_attributes1" ${bam_dir}/hg19/mapped_hg19_only/${sample}_only_hg19_sort.bam

setfattr -n user.comment -v "$new_attributes1" ${bam_dir}/hg19/mapped_hg19_only/${sample}_only_hg19_sort.bam.bai

done < $sample_file

##############################################################################################

########## 2nd call of pgMapstats.sh: unmapped_mm10 -> mapped to hg19 ##########

#

# command line arguments:

# argument 1: one item per line

# row 1: full path to input directory (containing sorted bam-files with index)

# row 2: full path to output directory

# row 3: experiment name - to be used as the file name for output

# row 4: full path to user directory

# argument 2: sample_file

# row 1 ff: full name of bam-file (including extension ".bam")

# last row: empty (\n)

printf "%s\n%s\n%s\n%s\n" "${bam_dir}/hg19/unmapped_mm10" "$mapstat_dir" "${experiment}_unmapped_mm10_mapped_hg19" "$user_dir" > ${user_dir}/arg21_mapstats

awk '{OFS="\t"} {print $1"_unmapped_mm10_sort.bam"}' $3 > ${user_dir}/arg22_mapstats

# run the script

printf "%s\n\n" "pgMapstats.sh mapped_hg19_unmapped_mm10" >> $log_long

pgMapstats.sh arg21_mapstats arg22_mapstats &>>$log_long

printf "%s\n\n" " " >> $log_short

cat "$user_dir/ver_mapstats" >> $log_short

cat ${user_dir}/arg2*_mapstats >> $log_short

rm ${user_dir}/arg2*_mapstats

rm "$user_dir/ver_mapstats"

min=`cat "${mapstat_dir}/${experiment}_unmapped_mm10_mapped_hg19.min_mapped_reads.txt"`

printf "%s\t%s\n" "min_mapped_reads unmapped_mm10_mapped_hg19:" $min >> $log_short

##################################################################################

########## 3rd call of pgBowtie1.sh: mapped_mm10 -> map to hg19 ############

#

# command line arguments:

# argument 1: one item per line

# row 1: full path to fastq directory

# row 2: full path to output-directory (bam- & sam-directory)

# row 3: full path to user directory; optional - if no directory is provided, the "output directory" will be used

# row 4: full path to bowtie1 genome

# row 5: additional tophat/bowtie2 parameters (optional)

# argument 2: list of fastq-files

# row 1 ff: one fastq-file name per row (root name, without extension '.fastq')

# define the command line variables

if [ ! -d "${bam_dir}/hg19/mapped_mm10" ]; then

mkdir -p ${bam_dir}/hg19/mapped_mm10

fi

printf "%s\n%s\n%s\n%s\n" "${bam_dir}/mm10/mapped_mm10" "${bam_dir}/hg19/mapped_mm10" "$user_dir" "$bowtie1_dir/hg19" > ${user_dir}/arg31_bowtie1

awk '{OFS="\t"} {print $1"_mapped_mm10"}' $3 > ${user_dir}/arg32_bowtie1

# run the script

printf "%s\n\n" "pgChIPseq_spikein_mouse.sh:hg19_mapped_mm10" >> $log_long

pgBowtie1.sh ${user_dir}/arg31_bowtie1 ${user_dir}/arg32_bowtie1

printf "%s\n" " " >> $log_short

cat "$user_dir/ver_bowtie1" >> $log_short

cat ${user_dir}/arg3*_bowtie1 >> $log_short

rm ${user_dir}/arg3*_bowtie1

rm "$user_dir/ver_bowtie1"

###########################################################################

########## 3rd call of pgMapstats.sh: mapped_mm10 -> map to hg19 ####

#

# command line arguments:

# argument 1: one item per line

# row 1: full path to input directory (containing sorted bam-files with index)

# row 2: full path to output directory

# row 3: experiment name - to be used as the file name for output

# row 4: full path to user directory

# argument 2: sample_file

# row 1 ff: full name of bam-file (including extension ".bam")

# last row: empty (\n)

printf "%s\n%s\n%s\n%s\n" "${bam_dir}/hg19/mapped_mm10" "$mapstat_dir" "${experiment}_mapped_mm10_mapped_hg19" "$user_dir" > ${user_dir}/arg31_mapstats

awk '{OFS="\t"} {print $1"_mapped_mm10_sort.bam"}' $3 > ${user_dir}/arg32_mapstats

# run the script

printf "%s\n\n" "pgMapstats.sh mapped_hg19_mapped_mm10" >> $log_long

pgMapstats.sh arg31_mapstats arg32_mapstats &>>$log_long

printf "%s\n\n" " " >> $log_short

cat "$user_dir/ver_mapstats" >> $log_short

cat ${user_dir}/arg3*_mapstats >> $log_short

rm ${user_dir}/arg3*_mapstats

rm "$user_dir/ver_mapstats"

min=`cat "${mapstat_dir}/${experiment}_mapped_mm10_mapped_hg19.min_mapped_reads.txt"`

printf "%s\t%s\n" "min_mapped_reads mapped_mm10_mapped_hg19:" $min >> $log_short

##################################################################################

##### establish fastq-files: mapped_mm10 -> unmapped_hg19 -> mapped_mm10 #####

#

if [ ! -d "${bam_dir}/mm10/unmapped_hg19" ]; then

mkdir -p ${bam_dir}/mm10/unmapped_hg19

fi

while read sample; do

samtools bam2fq -@ 5 -f 4 $bam_dir/hg19/mapped_mm10/${sample}_mapped_mm10_sort.bam > ${bam_dir}/mm10/unmapped_hg19/${sample}_mapped_mm10_unmapped_hg19.fastq # original: -@ 20

# add extended attributes

old_attributes=`getfattr -n user.comment $bam_dir/hg19/mapped_mm10/${sample}_mapped_mm10_sort.bam | sed '1d' | sed 's/user.comment=//' | sed 's/\"//g'`

date=`date +%y%m%d`

added_attributes1="<MOD>$VER_CHIPSEQ_SPIKEIN_MOUSE</MOD><DAT>$date</DAT><GEN>unmapped_to_mm10</GEN><FIL>$bam_dir/hg19/mapped_mm10/${sample}_mapped_mm10.bam</FIL>"

new_attributes1="${old_attributes}${added_attributes1}"

setfattr -n user.comment -v "$new_attributes1" ${bam_dir}/mm10/unmapped_hg19/${sample}_mapped_mm10_unmapped_hg19.fastq

done < $sample_file

################################################################################################

########## 4th call of pgBowtie1.sh: mm10_mapped -> hg19_unmapped -> map to mm10 ##########

#

# command line arguments:

# argument 1: one item per line

# row 1: full path to fastq directory

# row 2: full path to output-directory (bam- & sam-directory)

# row 3: full path to user directory; optional - if no directory is provided, the "output directory" will be used

# row 4: full path to bowtie1 genome

# row 5: additional tophat/bowtie2 parameters (optional)

# argument 2: list of fastq-files

# row 1 ff: one fastq-file name per row (root name, without extension '.fastq')

printf "%s\n%s\n%s\n%s\n" "${bam_dir}/mm10/unmapped_hg19" "${bam_dir}/mm10/unmapped_hg19" "$user_dir" "$bowtie1_dir/mm10" > ${user_dir}/arg41_bowtie1

awk '{OFS="\t"} {print $1"_mapped_mm10_unmapped_hg19"}' $3 > ${user_dir}/arg42_bowtie1

# run the script

printf "%s\n\n" "pgChIPseq_spikein_mouse.sh:mm10_mapped_mm10_unmapped_hg19" >> $log_long

pgBowtie1.sh ${user_dir}/arg41_bowtie1 ${user_dir}/arg42_bowtie1

printf "%s\n" " " >> $log_short

cat "$user_dir/ver_bowtie1" >> $log_short

cat ${user_dir}/arg4*_bowtie1 >> $log_short

rm ${user_dir}/arg4*_bowtie1

rm "$user_dir/ver_bowtie1"

##################################################################################################

##### establish bam-files: mapped_mm10_only = mm10_mapped -> hg19_unmapped -> mm10_mapped #####

#

if [ ! -d "${bam_dir}/mm10/mapped_mm10_only" ]; then

mkdir -p ${bam_dir}/mm10/mapped_mm10_only

fi

while read sample; do

samtools view -@ 5 -F 4 -O BAM -o ${bam_dir}/mm10/mapped_mm10_only/${sample}_only_mm10.bam ${bam_dir}/mm10/unmapped_hg19/${sample}_mapped_mm10_unmapped_hg19_sort.bam # original: -@ 20

samtools sort -@ 5 ${bam_dir}/mm10/mapped_mm10_only/${sample}_only_mm10.bam -o ${bam_dir}/mm10/mapped_mm10_only/${sample}_only_mm10_sort.bam # original: -@ 20

rm ${bam_dir}/mm10/mapped_mm10_only/${sample}_only_mm10.bam

samtools index ${bam_dir}/mm10/mapped_mm10_only/${sample}_only_mm10_sort.bam

mappedreads=`samtools idxstats ${bam_dir}/mm10/mapped_mm10_only/${sample}_only_mm10_sort.bam | grep -v "*" | awk '{s+=$3} END {print s}'`

unmappedreads=`samtools idxstats ${bam_dir}/mm10/mapped_mm10_only/${sample}_only_mm10_sort.bam | grep "*" | awk '{print $4}'`

total=`echo $((mappedreads+unmappedreads))`

# add extended attributes

old_attributes=`getfattr -n user.comment ${bam_dir}/mm10/unmapped_hg19/${sample}_mapped_mm10_unmapped_hg19_sort.bam | sed '1d' | sed 's/user.comment=//' | sed 's/\"//g'`

date=`date +%y%m%d`

added_attributes1="<MOD>$VER_CHIPSEQ_SPIKEIN_MOUSE</MOD><GEN>only_mapped_to_hg19</GEN><FIL>${bam_dir}/mm10/unmapped_hg19/${sample}_mapped_mm10_unmapped_hg19_sort.bam</FIL><NUM>$total</NUM><MAP>$mappedreads</MAP><UNM>$unmappedreads</UNM>"

new_attributes1="${old_attributes}${added_attributes1}"

setfattr -n user.comment -v "$new_attributes1" ${bam_dir}/mm10/mapped_mm10_only/${sample}_only_mm10_sort.bam

setfattr -n user.comment -v "$new_attributes1" ${bam_dir}/mm10/mapped_mm10_only/${sample}_only_mm10_sort.bam.bai

done < $sample_file

############################################################################################

########## 4th call of pgMapstats.sh: mm10_mapped -> hg19_unmapped -> map to mm10 ####

#

# command line arguments:

# argument 1: one item per line

# row 1: full path to input directory (containing sorted bam-files with index)

# row 2: full path to output directory

# row 3: experiment name - to be used as the file name for output

# row 4: full path to user directory

# argument 2: sample_file

# row 1 ff: full name of bam-file (including extension ".bam")

# last row: empty (\n)

printf "%s\n%s\n%s\n%s\n" "${bam_dir}/mm10/unmapped_hg19" "$mapstat_dir" "${experiment}_mapped_mm10_unmapped_hg19" "$user_dir" > ${user_dir}/arg41_mapstats

awk '{OFS="\t"} {print $1"_mapped_mm10_unmapped_hg19_sort.bam"}' $3 > ${user_dir}/arg42_mapstats

# run the script

printf "%s\n\n" "pgMapstats.sh mapped_hg19_mapped_mm10" >> $log_long

pgMapstats.sh arg41_mapstats arg42_mapstats &>>$log_long

printf "%s\n\n" " " >> $log_short

cat "$user_dir/ver_mapstats" >> $log_short

cat ${user_dir}/arg4*_mapstats >> $log_short

rm ${user_dir}/arg4*_mapstats

rm "$user_dir/ver_mapstats"

min=`cat "${mapstat_dir}/${experiment}_mapped_mm10_unmapped_hg19.min_mapped_reads.txt"`

printf "%s\t%s\n" "min_mapped_reads mapped_hg19_mapped_mm10:" $min >> $log_short

###############################################

########## end of the program ##########

end=`date +%s`

runtime=$((end-start))

printf "\n%s %s\n" "end:" `date +%d/%m/%Y,%H:%M:%S` >> $log_short

printf '%s %02dh:%02dm:%02ds\n' "Run duration: " $(($runtime/3600)) $(($runtime%3600/60)) $(($runtime%60)) >>$log_short

#!/bin/bash

VER_BEDGRAPH="pgBedgraph.sh_1.2.0"

#########################################

########## Description ##########

# functionality:

# reads list of bam-files

# randomly extracts indicated number of reads & writes corresponding output

# sorts the output bam-file

# writes bedgraph file for the shortened bam-file

#

# use:

# pgBedgraph.sh arg1 arg2 &>log.txt &; disown -h %1

#

# command line arguments:

# argument 1: one item per line

# row 1: full path to input directory (containing bam-files)

# row 2: full path to output directory (containing bedgraph-files)

# row 3: full path to user directory; optional - if no directory is provided, the "output directory" will be used

# row 4: genome length file (full path), containing in col1 the chromosome names & in col2 the number of nucleotids

# row 5: empty (\n)

# argument 2: sample_list

# each row: one sample

# col 1 ff: full name of bam-file (without extension ".bam")

# col 2 ff: number of reads to be used

# last row: empty (\n)

# retrieve parameters

bam_dir=`sed -n '1p' < $1`

bg_dir=`sed -n '2p' < $1`

user_dir=`sed -n '3p' < $1`

genome=`sed -n '4p' < $1`

samples=$2

# define sub for random number generation

get_seeded_random()

{

seed="$1"

openssl enc -aes-256-ctr -pass pass:"$seed" -nosalt </dev/zero 2>/dev/null

}

# do the analysis

while read name number; do

if [ -f "${bam_dir}/${name}.bam" ]; then

printf "calculate bedgraph for sample $name ...\n"

samtools view -@ 20 -H ${bam_dir}/${name}.bam > ${bam_dir}/${name}.header.txt

samtools view -@ 20 -F 4 ${bam_dir}/${name}.bam | grep -v '^@' - | shuf - --random-source=<(get_seeded_random 42) | head -n ${number} | cat ${bam_dir}/${name}.header.txt - | samtools view -@ 20 -bS - |samtools sort -@ 20 - -o ${bam_dir}/${name}_random${number}mapped.bam

samtools index ${bam_dir}/${name}_random${number}mapped.bam

rm ${bam_dir}/${name}.header.txt

bedtools genomecov -bg -g $genome -ibam ${bam_dir}/${name}_random${number}mapped.bam > ${bg_dir}/${name}_random${number}mapped.bedgraph

# set extended attributes

old_attributes=`getfattr -n user.comment ${bam_dir}/${name}.bam | sed '1d' | sed 's/user.comment=//' | sed 's/\"//g'`

date=`date +%y%m%d`

added_attributes="<MOD>$VER_BEDGRAPH</MOD><DAT>$date</DAT><FIL>${bam_dir}/${name}.bam</FIL><GEN>$genome</GEN>"

new_attributes="${old_attributes}${added_attributes}"

setfattr -n user.comment -v "$new_attributes" ${bg_dir}/${name}_random${number}mapped.bedgraph

fi

done < $samples

printf "%s\n" $VER_BEDGRAPH > $user_dir/ver_bedgraph
