# Supplementary material for: Association with TFIIIC limits MYCN localisation in hubs of active promoters and chromatin accumulation of non-phosphorylated RNA polymerase II
Source: eLife. 2024 Aug 23;13:RP94407. doi: 10.7554/eLife.94407 (PMC11343564; doi:10.7554/eLife.94407)
Supplement: Source code 2. [file elife-94407-code2.zip › Source code file 2.docx]

**Source code – Analysis of Hi-C/ Hi-ChIP data**

#!/bin/bash

helpFunction()

{

echo " "

echo " "

echo -e "--------------------------------------------------------------------------------------------------------"

echo " "

echo "Wrapper to fully analyse Hi-C/ Hi-ChIP data in paired-end sequencing on NextSeq -- Illumina"

echo "Please note that normalisation is not performed, expect if -s is determined"

echo "Please note that normalisation is ICE to all contact maps"

echo "script generate on 2021.11.03"

echo " "

echo "------------------------------------------------------ "

echo " "

echo -e "Usage: $0 \n-i folder input file -l observations to log -o output folder"

echo " "

echo "------------------------------------------------------ "

echo " "

echo -e "\t-i A file.txt with JUST ONE FILE (any file) PER LINE with PATH and extension. Ex: -i file.txt :inside: ˜/BaseSpace/foo/foo/SAMPLE/CA*/Files/*_L00X_RX_00X.fastq.gz"

echo -e "\t-l A file.txt explaining the experiment to be printed in the log file"

echo -e "\t-o Full path for output folder. Ex: /media/Data/foo/foo/foo"

echo -e "\t-c HiC-Pro configuration file name. Default: HiCPro_hg19_configuration.txt"

echo -e "\t-m Determines if inputs are to be merged based on the string that defines it as an input (CAN NOT CONTAIN 'Merged-'). Ex: IF yes :define: -m Input_HiC | ELSE: don't use -m "

echo -e "\t-p Number of threads to be used. Default: 110"

echo -e "\t-s Should files be normalised? NO = don't use -s | reads normalised = 'READS' | spike-in = 'SPIKE' "

echo -e "\t-g Name of the desired genome to be used as reference. Default: hg19"

echo -e "\t-t Chromosome size file for desired genome. Default: chrom_hg19.sizes"

echo -e "\t-r Genome restriction fragment file. Default: ~/DpnII_resfrag_hg19.bed"

echo -e "\t-v Resolution to build matrices. Ex: '2500 5000 10000' (...) | to not have .cool = don't use -f"

echo -e "\t-f Matrix resolution to be used for .cool format - has to be one used in -v parameter. Ex: 2500 | to not have .cool = don't use -f"

echo -e "\t-d Should duplicate interactions be removed? YES = 1 | NO = 0. Default: 1"

echo -e "\t-x Should multi mapped reads in interactions be removed? YES = 1 | NO = 0. Default: 1"

echo " "

echo " NEEDED ON PATH: BASEMOUNT & FASTQC & HiC-Pro & BOWTIE2 & CUTADAPT & HOMER & COOLER & COOLTOOLS & JUICERTOOLS "

echo " "

echo -e "--------------------------------------------------------------------------------------------------------"

echo " "

echo " "

exit 1 # Exit script after printing help

}

while getopts "i:l:o:c:m:p:s:g:t:r:v:f:d:x:" opt

do

case "$opt" in

i ) parameter1=$(<"$OPTARG") ;;

l ) parameter2=$(<"$OPTARG") ;;

o ) parameter3="$OPTARG" ;;

c ) parameter4="$OPTARG" ;;

m ) parameter5="$OPTARG" ;;

p ) parameter6="$OPTARG" ;;

s ) parameter7="$OPTARG" ;;

g ) parameter8="$OPTARG" ;;

t ) parameter9="$OPTARG" ;;

r ) parameter10="$OPTARG" ;;

v ) parameter11="$OPTARG" ;;

f ) parameter12="$OPTARG" ;;

d ) parameter13="$OPTARG" ;;

x ) parameter14="$OPTARG" ;;

? ) helpFunction ;; # Print helpFunction in case parameter is non-existent

esac

done

# default values

# Inserting default parameters in case none has been specified

#-c configuration file name

parameter4="${parameter4:-HiCPro_hg19_configuration.txt}"

#-p 100

parameter6="${parameter6:-110}"

#-s if READS or SPIKE is not is set

parameter7="${parameter7:-NADA}"

#-g genome name hg19

parameter8="${parameter8:-hg19}"

#-t chrom sizes (it uses relative pathway)

parameter9="${parameter9:-chrom_hg19.sizes}"

#-r resfrag full path

parameter10="${parameter10:-/foo/foo/Genomes_ref/bowtie2/Hsapiens/digested_ref/DpnII_resfrag_hg19.bed}"

#-d rm "duplicates" (1 Yes for HiC - 0 NO for HiChIP)

parameter13="${parameter13:-1}"

#-x rm multi mapped reads

parameter14="${parameter14:-1}"

#Setting number type to avoid issues

export LC_NUMERIC="en_US.UTF-8"

# Print helpFunction in case parameters are empty

if [ -z "$parameter1" ] || [ -z "$parameter2" ] || [ -z "$parameter3" ] || [ -z "$parameter4" ] || [ -z "$parameter6" ] || [ -z "$parameter8" ] || [ -z "$parameter9" ] || [ -z "$parameter10" ] || [ -z "$parameter13" ] || [ -z "$parameter14" ]

then

echo "Some or all of the parameters are empty";

helpFunction

fi

# changing name of parameter7 to hic-pro configuration file or denyin

if [ "$parameter7" = "SPIKE" ]; then

parameter7=HiCPro_mm10_configuration.txt

elif [ "$parameter7" = "READS" ] || [ "$parameter7" = "NADA" ] ; then

echo " "

else

echo "ERROR: cannot ID -s parameter"

echo " "

helpFunction

exit

fi

#OBSERVATIONS

#other variable - less used ones

#split_reads.py and utils_folder should have path updated:

utils_folder=/home/foo/Tools/HiC-Pro/bin/utils

juicer_folder=/home/foo/Tools/Juicer

#Number of chunks reads

chunks=10000000

#HiC-Pro parameter8 configuration file variables

csize=$(basename "$parameter9")

indexbtw2=/foo/foo/Genomes_ref/bowtie2/Hsapiens/

#Bridge set to 'GATCAGATTTGGGGATC' | 'GATCCCCAAATCTGATC'

#HiC-Pro mm10 configuration file variables

mref=mm10

msize=mm10.chrom.sizes

mresfrag=/foo/foo/Genomes_ref/bowtie2/Murine/reference_mm10/DpnII_resfrag_mm10.bed

mindexbtw2=/foo/foo/Genomes_ref/bowtie2/Murine/reference_mm10

# STARTING

today=$(date +"%d%m%Y")

echo "$(date) STARTING"

mkdir -p "$parameter3"/log_stats/fastqc/

echo "---EXPERIMENTAL OBSERVATION---" >> "$parameter3"/log_stats/log_script_HiCPro_"$today".txt

echo " " >> "$parameter3"/log_stats/log_script_HiCPro_"$today".txt

printf "$parameter2" >> "$parameter3"/log_stats/log_script_HiCPro_"$today".txt

echo " " >> "$parameter3"/log_stats/log_script_HiCPro_"$today".txt

echo "------------------------------" >> "$parameter3"/log_stats/log_script_HiCPro_"$today".txt

echo " " >> "$parameter3"/log_stats/log_script_HiCPro_"$today".txt

echo "---Folders---" >> "$parameter3"/log_stats/log_script_HiCPro_"$today".txt

echo " " >> "$parameter3"/log_stats/log_script_HiCPro_"$today".txt

printf "%s" $parameter1 >> "$parameter3"/log_stats/log_script_HiCPro_"$today".txt

echo " " >> "$parameter3"/log_stats/log_script_HiCPro_"$today".txt

echo "-------------" >> "$parameter3"/log_stats/log_script_HiCPro_"$today".txt

echo " " >> "$parameter3"/log_stats/log_script_HiCPro_"$today".txt

echo "---Options used---" >> "$parameter3"/log_stats/log_script_HiCPro_"$today".txt

echo " " >> "$parameter3"/log_stats/log_script_HiCPro_"$today".txt

echo -e " o $parameter3 \n c $parameter4 \n m $parameter5 \n p $parameter6 \n s $parameter7 \n g $parameter8 \n t $parameter9 \n r $parameter10 \n v $parameter11 \n f $parameter12 \n d $parameter13" >> "$parameter3"/log_stats/log_script_HiCPro_"$today".txt

echo " " >> "$parameter3"/log_stats/log_script_HiCPro_"$today".txt

echo "------------------" >> "$parameter3"/log_stats/log_script_HiCPro_"$today".txt

echo " " >> "$parameter3"/log_stats/log_script_HiCPro_"$today".txt

echo "---HiC-Pro "$parameter8" configuration---" >> "$parameter3"/log_stats/log_script_HiCPro_"$today".txt

echo " " >> "$parameter3"/log_stats/log_script_HiCPro_"$today".txt

########## DO NOT CHANGE BELOW: HiC-Pro configuration file for "$parameter8"

echo "# Please change the variable settings below if necessary

#########################################################################

## Paths and Settings - Do not edit !

#########################################################################

TMP_DIR = tmp

LOGS_DIR = logs

BOWTIE2_OUTPUT_DIR = bowtie_results

MAPC_OUTPUT = hic_results

RAW_DIR = rawdata

#######################################################################

## SYSTEM - PBS - Start Editing Here !!

#######################################################################

N_CPU = "$parameter6"

LOGFILE = hicpro.log

JOB_NAME = HiC_HiCPro_PE_full_analysis

JOB_MEM = 500gb

JOB_WALLTIME =

JOB_QUEUE =

JOB_MAIL =

#########################################################################

## Data

#########################################################################

PAIR1_EXT = _R1

PAIR2_EXT = _R2

#######################################################################

## Alignment options

#######################################################################

FORMAT = phred33

MIN_MAPQ = 0

BOWTIE2_IDX_PATH = "$indexbtw2"

BOWTIE2_GLOBAL_OPTIONS = --very-sensitive -L 25 --score-min L,-0.6,-0.2 --end-to-end --reorder -N 1

BOWTIE2_LOCAL_OPTIONS = --very-sensitive -L 15 --score-min L,-0.6,-0.2 --end-to-end --reorder -N 1

#######################################################################

## Annotation files

#######################################################################

REFERENCE_GENOME = "$parameter8"

GENOME_SIZE = "$csize"

#GENOME_SIZE = chrom_hg19.sizes

#######################################################################

## Allele specific

#######################################################################

ALLELE_SPECIFIC_SNP = " > "$parameter3"/log_stats/"$parameter4"

echo "

#######################################################################

## Digestion Hi-C

#######################################################################

#GENOME_FRAGMENT = HindIII_resfrag_hg19.bed

#LIGATION_SITE = AAGCTAGCTT

GENOME_FRAGMENT = "$parameter10"

LIGATION_SITE = GATCAGATTTGGGGATC,GATCAGATTTGGGGATCGATCAGATTTGGGGATC,GATCAGATTTGGGGATCGATCAGATTTGGGGATCGATCAGATTTGGGGATC,GATCCCCAAATCTGATC,GATCCCCAAATCTGATCGATCCCCAAATCTGATC,GATCCCCAAATCTGATCGATCCCCAAATCTGATCGATCCCCAAATCTGATC,GATCAGATTTGGGGATCGATCCCCAAATCTGATC,GATCCCCAAATCTGATCGATCAGATTTGGGGATC,GATCAGATTTGGGGATCGATCCCCAAATCTGATCGATCAGATTTGGGGATC,GATCAGATTTGGGGATCGATCCCCAAATCTGATCGATCCCCAAATCTGATC,GATCCCCAAATCTGATCGATCAGATTTGGGGATCGATCCCCAAATCTGATC,GATCCCCAAATCTGATCGATCAGATTTGGGGATCGATCAGATTTGGGGATC

MIN_FRAG_SIZE =

MAX_FRAG_SIZE =

MIN_INSERT_SIZE =

MAX_INSERT_SIZE =

#MIN_FRAG_SIZE = 100

#MAX_FRAG_SIZE = 100000

#MIN_INSERT_SIZE = 100

#MAX_INSERT_SIZE = 600

#######################################################################

## Hi-C processing

#######################################################################

MIN_CIS_DIST =

GET_ALL_INTERACTION_CLASSES = 1

GET_PROCESS_SAM = 1

RM_SINGLETON = 1

RM_MULTI = "$parameter14"

RM_DUP = "$parameter13"

#######################################################################

## Contact Maps

#######################################################################

BIN_SIZE = "$parameter11"

MATRIX_FORMAT = upper

#######################################################################

## ICE Normalization

#######################################################################

MAX_ITER = 100

FILTER_LOW_COUNT_PERC = 0.02

FILTER_HIGH_COUNT_PERC = 0

EPS = 0.1

" >> "$parameter3"/log_stats/"$parameter4"

##### DO NOT CHANGE ABOVE ######

printf "$parameter3"/log_stats/"$parameter4" >> "$parameter3"/log_stats/log_script_HiCPro_"$today".txt

echo " " >> "$parameter3"/log_stats/log_script_HiCPro_"$today".txt

echo "-----------------------------------------" >> "$parameter3"/log_stats/log_script_HiCPro_"$today".txt

echo " " >> "$parameter3"/log_stats/log_script_HiCPro_"$today".txt

if [ "$parameter7" = "NADA" ]; then

echo "-----HiC-Pro normalisation-----" >> "$parameter3"/log_stats/log_script_HiCPro_"$today".txt

echo " " >> "$parameter3"/log_stats/log_script_HiCPro_"$today".txt

echo " NO normalisation" >> "$parameter3"/log_stats/log_script_HiCPro_"$today".txt

echo " " >> "$parameter3"/log_stats/log_script_HiCPro_"$today".txt

echo "-------------------------------" >> "$parameter3"/log_stats/log_script_HiCPro_"$today".txt

echo " " >> "$parameter3"/log_stats/log_script_HiCPro_"$today".txt

elif [ "$parameter7" = "READS" ]; then

echo "-----HiC-Pro normalisation-----" >> "$parameter3"/log_stats/log_script_HiCPro_"$today".txt

echo " " >> "$parameter3"/log_stats/log_script_HiCPro_"$today".txt

echo " Reads normalisation: .allValidPairs normalisation" >> "$parameter3"/log_stats/log_script_HiCPro_"$today".txt

echo " " >> "$parameter3"/log_stats/log_script_HiCPro_"$today".txt

echo "-------------------------------" >> "$parameter3"/log_stats/log_script_HiCPro_"$today".txt

echo " " >> "$parameter3"/log_stats/log_script_HiCPro_"$today".txt

elif [ "$parameter7" = "HiCPro_mm10_configuration.txt" ]; then

echo "-----HiC-Pro normalisation-----" >> "$parameter3"/log_stats/log_script_HiCPro_"$today".txt

echo " " >> "$parameter3"/log_stats/log_script_HiCPro_"$today".txt

echo " Spike-in normalisation: .allValidPairs mm10 normalisation" >> "$parameter3"/log_stats/log_script_HiCPro_"$today".txt

echo " " >> "$parameter3"/log_stats/log_script_HiCPro_"$today".txt

echo "-------------------------------" >> "$parameter3"/log_stats/log_script_HiCPro_"$today".txt

########## DO NOT CHANGE BELOW: HiC-Pro configuration file for mm10

echo "# Please change the variable settings below if necessary

#########################################################################

## Paths and Settings - Do not edit !

#########################################################################

TMP_DIR = tmp

LOGS_DIR = logs

BOWTIE2_OUTPUT_DIR = bowtie_results

MAPC_OUTPUT = hic_results

RAW_DIR = rawdata

#######################################################################

## SYSTEM - PBS - Start Editing Here !!

#######################################################################

N_CPU = "$parameter6"

LOGFILE = hicpro.log

JOB_NAME = HiC_HiCPro_mice_PE_full_analysis

JOB_MEM = 500gb

JOB_WALLTIME =

JOB_QUEUE =

JOB_MAIL =

#########################################################################

## Data

#########################################################################

PAIR1_EXT = _R1

PAIR2_EXT = _R2

#######################################################################

## Alignment options

#######################################################################

FORMAT = phred33

MIN_MAPQ = 0

BOWTIE2_IDX_PATH = "$mindexbtw2"

BOWTIE2_GLOBAL_OPTIONS = --very-sensitive -L 25 --score-min L,-0.6,-0.2 --end-to-end --reorder -N 1

BOWTIE2_LOCAL_OPTIONS = --very-sensitive -L 15 --score-min L,-0.6,-0.2 --end-to-end --reorder -N 1

#######################################################################

## Annotation files

#######################################################################

REFERENCE_GENOME = "$mref"

GENOME_SIZE = "$mcsize"

#GENOME_SIZE = chrom_hg19.sizes

#######################################################################

## Allele specific

#######################################################################

ALLELE_SPECIFIC_SNP = " > "$parameter3"/log_stats/"$parameter7"

echo "

#######################################################################

## Digestion Hi-C

#######################################################################

#GENOME_FRAGMENT = HindIII_resfrag_hg19.bed

#LIGATION_SITE = AAGCTAGCTT

GENOME_FRAGMENT = "$mresfrag"

LIGATION_SITE = GATCAGATTTGGGGATC,GATCAGATTTGGGGATCGATCAGATTTGGGGATC,GATCAGATTTGGGGATCGATCAGATTTGGGGATCGATCAGATTTGGGGATC,GATCCCCAAATCTGATC,GATCCCCAAATCTGATCGATCCCCAAATCTGATC,GATCCCCAAATCTGATCGATCCCCAAATCTGATCGATCCCCAAATCTGATC,GATCAGATTTGGGGATCGATCCCCAAATCTGATC,GATCCCCAAATCTGATCGATCAGATTTGGGGATC,GATCAGATTTGGGGATCGATCCCCAAATCTGATCGATCAGATTTGGGGATC,GATCAGATTTGGGGATCGATCCCCAAATCTGATCGATCCCCAAATCTGATC,GATCCCCAAATCTGATCGATCAGATTTGGGGATCGATCCCCAAATCTGATC,GATCCCCAAATCTGATCGATCAGATTTGGGGATCGATCAGATTTGGGGATC

MIN_FRAG_SIZE =

MAX_FRAG_SIZE =

MIN_INSERT_SIZE =

MAX_INSERT_SIZE =

#MIN_FRAG_SIZE = 100

#MAX_FRAG_SIZE = 100000

#MIN_INSERT_SIZE = 100

#MAX_INSERT_SIZE = 600

#######################################################################

## Hi-C processing

#######################################################################

MIN_CIS_DIST =

GET_ALL_INTERACTION_CLASSES = 1

GET_PROCESS_SAM = 1

RM_SINGLETON = 1

RM_MULTI = "$parameter14"

RM_DUP = "$parameter13"

#######################################################################

## Contact Maps

#######################################################################

BIN_SIZE = "$parameter11"

MATRIX_FORMAT = upper

#######################################################################

## ICE Normalization

#######################################################################

MAX_ITER = 100

FILTER_LOW_COUNT_PERC = 0.02

FILTER_HIGH_COUNT_PERC = 0

EPS = 0.1

" >> "$parameter3"/log_stats/"$parameter7"

##### DO NOT CHANGE ABOVE ######

printf "$parameter3"/log_stats/"$parameter7" >> "$parameter3"/log_stats/log_script_HiCPro_"$today".txt

echo " " >> "$parameter3"/log_stats/log_script_HiCPro_"$today".txt

echo "--------------------------------" >> "$parameter3"/log_stats/log_script_HiCPro_"$today".txt

echo " " >> "$parameter3"/log_stats/log_script_HiCPro_"$today".txt

fi

echo "---START TIME---" >> "$parameter3"/log_stats/log_script_HiCPro_"$today".txt

echo " " >> "$parameter3"/log_stats/log_script_HiCPro_"$today".txt

date >> "$parameter3"/log_stats/log_script_HiCPro_"$today".txt

echo " " >> "$parameter3"/log_stats/log_script_HiCPro_"$today".txt

echo "----------------" >> "$parameter3"/log_stats/log_script_HiCPro_"$today".txt

echo " " >> "$parameter3"/log_stats/log_script_HiCPro_"$today".txt

echo " " >> "$parameter3"/log_stats/log_script_HiCPro_"$today".txt

echo "-- $(date) -- Transfering files from BaseSpace" | tee -a "$parameter3"/log_stats/log_script_HiCPro_"$today".txt

echo " " >> "$parameter3"/log_stats/log_script_HiCPro_"$today".txt

# Preparing Files

for sample in $parameter1; do

mkdir -p "$parameter3"/raw_data/$(basename "$sample" | sed "s/_.*//")

echo "cp files in $(basename "$sample" | sed "s/_.*//")" >> "$parameter3"/log_stats/log_script_HiCPro_"$today".txt

cp $(dirname "$sample")/*.fastq.gz "$parameter3"/raw_data/$(basename "$sample" | sed "s/_.*//")

cd "$parameter3"/raw_data/$(basename "$sample" | sed "s/_.*//")

gunzip *.gz

#cat files to to do QC analysis

cat *_R1* > $(basename "$sample" | sed "s/_.*/_R1.fastq/")

cat *_R2* > $(basename "$sample" | sed "s/_.*/_R2.fastq/")

echo "going to next file"

rm -f *L001* *L002* *L003* *L004*

done

#preparing bridges to feed as contaminants (IT IS NOT) to have a overview in fastqc:

echo -e "bridge1\tGATCAGATTTGGGGATC\nbridge2\tGATCAGATTTGGGGATCGATCAGATTTGGGGATC\nbridge3\tGATCAGATTTGGGGATCGATCAGATTTGGGGATCGATCAGATTTGGGGATC\nbridge1_rev\tGATCCCCAAATCTGATC\nbridge2_rev\tGATCCCCAAATCTGATCGATCCCCAAATCTGATC\nbridge3_rev\tGATCCCCAAATCTGATCGATCCCCAAATCTGATCGATCCCCAAATCTGATC" > "$parameter3"/log_stats/fastqc/qc_bridges.txt

echo " " >> "$parameter3"/log_stats/log_script_HiCPro_"$today".txt

echo "-- $(date) -- Counting reads number and doing QC (FASTQC)" | tee -a "$parameter3"/log_stats/log_script_HiCPro_"$today".txt

echo " " >> "$parameter3"/log_stats/log_script_HiCPro_"$today".txt

cd "$parameter3"

for file in $(find "$parameter3"/raw_data -type f -name '*.fastq'); do

echo "-- $(date) -- counting reads number on $file" >> "$parameter3"/log_stats/log_script_HiCPro_"$today".txt

echo " " >> "$parameter3"/log_stats/log_script_HiCPro_"$today".txt

{ echo "$file" ; grep "^@" "$file" | wc -l ; } | cat >> "$parameter3"/log_stats/log_script_HiCPro_"$today".txt

echo " " >> "$parameter3"/log_stats/log_script_HiCPro_"$today".txt

echo "-- $(date) -- fastqc on $file" >> "$parameter3"/log_stats/log_script_HiCPro_"$today".txt

fastqc --outdir "$parameter3"/log_stats/fastqc --contaminants "$parameter3"/log_stats/fastqc/qc_bridges.txt --threads 6 "$file"

echo " " >> "$parameter3"/log_stats/log_script_HiCPro_"$today".txt

done

#Are Input samples to be merged? - it may be advantageous for HiChIP

if [ -z "$parameter5" ]; then

echo " " >> "$parameter3"/log_stats/log_script_HiCPro_"$today".txt

echo "Null -m parameter. Inputs will be used as it is"

echo "Inputs will not be merged. It will be used as it is" >> "$parameter3"/log_stats/log_script_HiCPro_"$today".txt

echo " " >> "$parameter3"/log_stats/log_script_HiCPro_"$today".txt

else

echo " " >> "$parameter3"/log_stats/log_script_HiCPro_"$today".txt

echo "Detected -m parameter $parameter5 . Inputs will be merged in "$parameter3"/Merged_input_HiC"

echo "Merging inputs by string $parameter5 . Merged files in "$parameter3"/Merged_input_HiC" >> "$parameter3"/log_stats/log_script_HiCPro_"$today".txt

echo " " >> "$parameter3"/log_stats/log_script_HiCPro_"$today".txt

mkdir -p "$parameter3"/raw_data/Merged-HiC-input

cd "$parameter3"/raw_data/Merged-HiC-input

#all inputs

merging=$(find "$parameter3"/raw_data/ -type f -iname "*$parameter5*" | grep "R1.fastq")

cat $(echo "$merging" | tr '\n' ' ') > Merged-HiC-input_R1.fastq

merging2=$(find "$parameter3"/raw_data/ -type f -iname "*$parameter5*" | grep "R2.fastq")

cat $(echo "$merging2" | tr '\n' ' ') > Merged-HiC-input_R2.fastq

#to remove all, but the recently merged, input files

find "$parameter3"/raw_data/ -type d -iname "*$parameter5*" ! -iname "Merged-HiC-input*" -exec rm -r {} +

fi

#spliting reads R1 or R2 in 10M chunks to speed up bowtie2 mapping

echo " " >> "$parameter3"/log_stats/log_script_HiCPro_"$today".txt

echo "-- $(date) -- Spliting reads in $chunks chunks to speed up HiC-Pro (bowtie2) mapping" | tee -a "$parameter3"/log_stats/log_script_HiCPro_"$today".txt

echo " " >> "$parameter3"/log_stats/log_script_HiCPro_"$today".txt

if [ -z "$parameter5" ]; then

#no merge

for sample in $parameter1; do

cd "$parameter3"/raw_data/$(basename "$sample" | sed "s/_.*//")

"$utils_folder"/split_reads.py *_R1.fastq --results_folder "$parameter3"/raw_data/$(basename "$sample" | sed "s/_.*//") --nreads "$chunks"

"$utils_folder"/split_reads.py *_R2.fastq --results_folder "$parameter3"/raw_data/$(basename "$sample" | sed "s/_.*//") --nreads "$chunks"

rm $(basename "$sample" | sed "s/_.*/_R1.fastq/") $(basename "$sample" | sed "s/_.*/_R2.fastq/")

echo "going to next file"

done

else

#merged inputs

for sample in $(grep -vi "$parameter5" "$parameter1"); do

cd "$parameter3"/raw_data/$(basename "$sample" | sed "s/_.*//")

"$utils_folder"/split_reads.py *_R1.fastq --results_folder "$parameter3"/raw_data/$(basename "$sample" | sed "s/_.*//") --nreads "$chunks"

"$utils_folder"/split_reads.py *_R2.fastq --results_folder "$parameter3"/raw_data/$(basename "$sample" | sed "s/_.*//") --nreads "$chunks"

rm $(basename "$sample" | sed "s/_.*/_R1.fastq/") $(basename "$sample" | sed "s/_.*/_R2.fastq/")

echo "going to next file"

done

cd "$parameter3"/raw_data/Merged-HiC-input

"$utils_folder"/split_reads.py Merged-HiC-input_R1.fastq --results_folder "$parameter3"/raw_data/Merged-HiC-input --nreads "$chunks"

"$utils_folder"/split_reads.py Merged-HiC-input_R2.fastq --results_folder "$parameter3"/raw_data/Merged-HiC-input --nreads "$chunks"

rm Merged-HiC-input_R1.fastq Merged-HiC-input_R2.fastq

fi

#from now on the parameter 5 (merge or not inputs) will not require further attention

#how normalisation will be perfomed: nothing or .allValidPairs number or mm10 spikein ratio?

if [ "$parameter7" = "NADA" ]; then

echo " " >> "$parameter3"/log_stats/log_script_HiCPro_"$today".txt

echo "--------------------------------------------------------------------" >> "$parameter3"/log_stats/log_script_HiCPro_"$today".txt

echo " " >> "$parameter3"/log_stats/log_script_HiCPro_"$today".txt

echo "-- $(date) -- HiC-Pro all steps" | tee -a "$parameter3"/log_stats/log_script_HiCPro_"$today".txt

echo " " >> "$parameter3"/log_stats/log_script_HiCPro_"$today".txt

echo "--------------------------------------------------------------------" >> "$parameter3"/log_stats/log_script_HiCPro_"$today".txt

echo " " >> "$parameter3"/log_stats/log_script_HiCPro_"$today".txt

HiC-Pro -i "$parameter3"/raw_data -o "$parameter3"/HiCPro_"$parameter8"_"$today" -c "$parameter3"/log_stats/"$parameter4" 2>> "$parameter3"/log_stats/log_script_HiCPro_"$today".txt

elif [ "$parameter7" = "READS" ]; then

# .allValidPairs number normalisation

#HiC-Pro

echo " " >> "$parameter3"/log_stats/log_script_HiCPro_"$today".txt

echo "--------------------------------------------------------------------" >> "$parameter3"/log_stats/log_script_HiCPro_"$today".txt

echo " " >> "$parameter3"/log_stats/log_script_HiCPro_"$today".txt

echo "-- $(date) -- HiC-Pro step wise from map to normalisation point (.allValidPairs)" | tee -a "$parameter3"/log_stats/log_script_HiCPro_"$today".txt

echo " " >> "$parameter3"/log_stats/log_script_HiCPro_"$today".txt

echo "--------------------------------------------------------------------" >> "$parameter3"/log_stats/log_script_HiCPro_"$today".txt

echo " " >> "$parameter3"/log_stats/log_script_HiCPro_"$today".txt

HiC-Pro -i "$parameter3"/raw_data -o "$parameter3"/HiCPro_"$parameter8"_"$today" -c "$parameter3"/log_stats/"$parameter4" -s mapping -s proc_hic -s quality_checks -s merge_persample 2>> "$parameter3"/log_stats/log_script_HiCPro_"$today".txt

echo "--------------------------------------------------------------------" >> "$parameter3"/log_stats/log_script_HiCPro_"$today".txt

echo " " >> "$parameter3"/log_stats/log_script_HiCPro_"$today".txt

echo "-- $(date) -- Samples will be normalised by total number of .allValidPairs" | tee -a "$parameter3"/log_stats/log_script_HiCPro_"$today".txt

echo " " >> "$parameter3"/log_stats/log_script_HiCPro_"$today".txt

echo "--------------------------------------------------------------------" >> "$parameter3"/log_stats/log_script_HiCPro_"$today".txt

echo " " >> "$parameter3"/log_stats/log_script_HiCPro_"$today".txt

for sample in $(find "$parameter3"/HiCPro_"$parameter8"_"$today" -type f -name '*.allValidPairs'); do

cd $(dirname "$sample")

echo "$(basename "$sample" | sed "s/_.*//") number of interactions:" >> "$parameter3"/log_stats/log_script_HiCPro_"$today".txt

wc -l "$sample" | cut -f1 -d" " | tee -a "$parameter3"/log_stats/interactions_number_"$parameter8"_TEMPORARIO.txt >> "$parameter3"/log_stats/log_script_HiCPro_"$today".txt

echo " " >> "$parameter3"/log_stats/log_script_HiCPro_"$today".txt

done

min1=$(sort -n "$parameter3"/log_stats/interactions_number_"$parameter8"_TEMPORARIO.txt | head -1)

echo "--------------" >> "$parameter3"/log_stats/log_script_HiCPro_"$today".txt

echo " " >> "$parameter3"/log_stats/log_script_HiCPro_"$today".txt

echo "Samples will be normalised by $min1 interactions" >> "$parameter3"/log_stats/log_script_HiCPro_"$today".txt

echo " " >> "$parameter3"/log_stats/log_script_HiCPro_"$today".txt

echo "--------------" >> "$parameter3"/log_stats/log_script_HiCPro_"$today".txt

seeding=0

for sample in $(find "$parameter3"/HiCPro_"$parameter8"_"$today" -type f -name '*.allValidPairs'); do

cd $(dirname "$sample")

echo "working on $(basename "$sample")"

#function to store seed

get_seeded_random()

{

pickrandom="$1"

openssl enc -aes-256-ctr -pass pass:"$pickrandom" -nosalt \

</dev/zero 2>/dev/null

}

# automatic seed generation, which "date" HourMinuteSecondDayMonthYear + "seeding" out and within the loop makes each seed unique

seeding=$(expr $(date +"%H%M%S%d%m%Y") + "$seeding")

echo " " >> "$parameter3"/log_stats/log_script_HiCPro_"$today".txt

echo -e "$sample \n seed $seeding" >> "$parameter3"/log_stats/log_script_HiCPro_"$today".txt

sort -k2,2V -k3,3n "$sample" | shuf --random-source=<(get_seeded_random "$seeding") | head -n "$min1" | sort -k2,2V -k3,3n > $(basename "$sample" | sed "s/.allValidPairs/_norm.allValidPairs/")

#changing file extension of not normalised .allValidPairs to avoid conflicts in the next HiC-Pro call

mv "$sample" $(basename "$sample" | sed "s/.allValidPairs/.notnormpairs/")

done

echo " " >> "$parameter3"/log_stats/log_script_HiCPro_"$today".txt

echo "--------------" >> "$parameter3"/log_stats/log_script_HiCPro_"$today".txt

echo " " >> "$parameter3"/log_stats/log_script_HiCPro_"$today".txt

# Continuing with HiC-Pro

echo "--------------------------------------------------------------------" >> "$parameter3"/log_stats/log_script_HiCPro_"$today".txt

echo " " >> "$parameter3"/log_stats/log_script_HiCPro_"$today".txt

echo "-- $(date) -- HiC-Pro step wise from normalisation point (.allValidPairs) to matrix" | tee -a "$parameter3"/log_stats/log_script_HiCPro_"$today".txt

echo " " >> "$parameter3"/log_stats/log_script_HiCPro_"$today".txt

echo "--------------------------------------------------------------------" >> "$parameter3"/log_stats/log_script_HiCPro_"$today".txt

echo " " >> "$parameter3"/log_stats/log_script_HiCPro_"$today".txt

HiC-Pro -i "$parameter3"/hic_results/data/ -o "$parameter3"/HiCPro_"$parameter8"_"$today" -c "$parameter3"/log_stats/"$parameter4" -s build_contact_maps -s ice_norm 2>> "$parameter3"/log_stats/log_script_HiCPro_"$today".txt

elif [ "$parameter7" = "HiCPro_mm10_configuration.txt" ]; then

# .allValidPairs spike in normalisation

echo " " >> "$parameter3"/log_stats/log_script_HiCPro_"$today".txt

echo "--------------------------------------------------------------------" >> "$parameter3"/log_stats/log_script_HiCPro_"$today".txt

echo " " >> "$parameter3"/log_stats/log_script_HiCPro_"$today".txt

echo "-- $(date) -- HiC-Pro step wise from map to normalisation point (.allValidPairs)" | tee -a "$parameter3"/log_stats/log_script_HiCPro_"$today".txt

echo " " >> "$parameter3"/log_stats/log_script_HiCPro_"$today".txt

echo "--------------------------------------------------------------------" >> "$parameter3"/log_stats/log_script_HiCPro_"$today".txt

echo " " >> "$parameter3"/log_stats/log_script_HiCPro_"$today".txt

HiC-Pro -i "$parameter3"/raw_data -o "$parameter3"/HiCPro_"$parameter8"_"$today" -c "$parameter3"/log_stats/"$parameter4" -s mapping -s proc_hic -s quality_checks -s merge_persample 2>> "$parameter3"/log_stats/log_script_HiCPro_"$today".txt

echo "--------------------------------------------------------------------" >> "$parameter3"/log_stats/log_script_HiCPro_"$today".txt

echo " " >> "$parameter3"/log_stats/log_script_HiCPro_"$today".txt

echo "-- $(date) -- Samples will be normalised by the ratio of mm10 with least .allValidPairs/ its mm10 .allValidPairs x hg19 .allValidPairs" | tee -a "$parameter3"/log_stats/log_script_HiCPro_"$today".txt

echo " " >> "$parameter3"/log_stats/log_script_HiCPro_"$today".txt

echo "--------------------------------------------------------------------" >> "$parameter3"/log_stats/log_script_HiCPro_"$today".txt

echo " " >> "$parameter3"/log_stats/log_script_HiCPro_"$today".txt

echo " number in .allValidPairs before spike-in normalisation" | tee -a "$parameter3"/log_stats/log_script_HiCPro_"$today".txt

echo " " >> "$parameter3"/log_stats/log_script_HiCPro_"$today".txt

for sample in $(find "$parameter3"/HiCPro_"$parameter8"_"$today" -type f -name '*.allValidPairs'); do

cd $(dirname "$sample")

echo "$(basename "$sample" | sed "s/_.*//")" >> "$parameter3"/log_stats/log_script_HiCPro_"$today".txt

wc -l "$sample" | cut -f1 -d" " >> "$parameter3"/log_stats/log_script_HiCPro_"$today".txt

echo " " >> "$parameter3"/log_stats/log_script_HiCPro_"$today".txt

done

echo "----------------" >> "$parameter3"/log_stats/log_script_HiCPro_"$today".txt

#HiCpro map mm10 to generate spike-in .allValidPairs

HiC-Pro -i "$parameter3"/raw_data -o "$parameter3"/HiCPro_mm10_"$today" -c "$parameter3"/log_stats/"$parameter7" -s mapping -s proc_hic -s quality_checks -s merge_persample 2>> "$parameter3"/log_stats/log_script_HiCPro_"$today".txt

echo " " >> "$parameter3"/log_stats/log_script_HiCPro_"$today".txt

echo " number of interactions in spike-in and after spike-in removal" | tee -a "$parameter3"/log_stats/log_script_HiCPro_"$today".txt

echo " " >> "$parameter3"/log_stats/log_script_HiCPro_"$today".txt

#remove mm10 .allValidPairs from hg19 .allValidPairs and discovering mm10 .allValidPairs with less interactions to use as min

for sample in $(find "$parameter3"/HiCPro_"$parameter8"_"$today" -type f -name '*.allValidPairs'); do

cd $(dirname "$sample")

awk 'FNR==NR{a[$1];next};!($1 in a)' $(echo "$sample" | sed "s#HiCPro_"$parameter8"_"$today"#HiCPro_mm10_"$today"#") "$sample" > $(echo "$sample" | sed "s/.allValidPairs/_"$parameter8"_TEMPORARIO.allValidPairs/")

echo "$(basename "$sample" | sed "s/_.*//") number of mm10 interactions:" >> "$parameter3"/log_stats/log_script_HiCPro_"$today".txt

wc -l $(echo "$sample" | sed "s#HiCPro_"$parameter8"_"$today"#HiCPro_mm10_"$today"#") | cut -f1 -d" " | tee -a "$parameter3"/log_stats/interactions_number_mm10_TEMPORARIO.txt >> "$parameter3"/log_stats/log_script_HiCPro_"$today".txt

echo " " >> "$parameter3"/log_stats/log_script_HiCPro_"$today".txt

echo "$(basename "$sample" | sed "s/_.*//") number of "$parameter8" valid pairs after mm10 interactions removal:" >> "$parameter3"/log_stats/log_script_HiCPro_"$today".txt

wc -l $(echo "$sample" | sed "s/.allValidPairs/_"$parameter8"_TEMPORARIO.allValidPairs/") | cut -f1 -d" " >> "$parameter3"/log_stats/log_script_HiCPro_"$today".txt

echo " " >> "$parameter3"/log_stats/log_script_HiCPro_"$today".txt

done

echo "----------------" >> "$parameter3"/log_stats/log_script_HiCPro_"$today".txt

min1=$(sort -n "$parameter3"/log_stats/interactions_number_mm10_TEMPORARIO.txt | head -1)

echo " " >> "$parameter3"/log_stats/log_script_HiCPro_"$today".txt

echo "-- Samples will be normalised by the ratio with $min1 mice interactions" >> "$parameter3"/log_stats/log_script_HiCPro_"$today".txt

echo " " >> "$parameter3"/log_stats/log_script_HiCPro_"$today".txt

#HiCpro hg19 .allValidPairs spikein normalise: ratio of its mm10 .allValidPairs/ mm10 with least .allValidPairs x hg19 .allValidPairs

seeding=0

for sample in $(find "$parameter3"/HiCPro_"$parameter8"_"$today" -type f -name '*.allValidPairs' ! -name "*TEMPORARIO*"); do

cd $(dirname "$sample")

echo "working on $(basename "$sample")"

spikem=$(wc -l $(echo "$sample" | sed "s#HiCPro_"$parameter8"_"$today"#HiCPro_mm10_"$today"#") | cut -f1 -d" " )

genomeh=$(wc -l $(echo "$sample" | sed "s/.allValidPairs/_"$parameter8"_TEMPORARIO.allValidPairs/") | cut -f1 -d" ")

ratio=$(awk "BEGIN { a = $min1; b = $spikem; d = $genomeh; printf \"%.f\n\", (a / b * d) }")

#function to store seed

get_seeded_random()

{

pickrandom="$1"

openssl enc -aes-256-ctr -pass pass:"$pickrandom" -nosalt \

</dev/zero 2>/dev/null

}

# automatic seed generation, which "date" HourMinuteSecondDayMonthYear + "seeding" out and within the loop makes each seed unique

seeding=$(expr $(date +"%H%M%S%d%m%Y") + "$seeding")

echo " " >> "$parameter3"/log_stats/log_script_HiCPro_"$today".txt

echo -e " $sample \n seed $seeding \n N reads $ratio" >> "$parameter3"/log_stats/log_script_HiCPro_"$today".txt

echo " " >> "$parameter3"/log_stats/log_script_HiCPro_"$today".txt

sort -k2,2V -k3,3n $(echo "$sample" | sed "s/.allValidPairs/_"$parameter8"_TEMPORARIO.allValidPairs/") | shuf --random-source=<(get_seeded_random "$seeding") | head -n "$ratio" | sort -k2,2V -k3,3n > $(basename "$sample" | sed "s/.allValidPairs/_snorm.allValidPairs/")

#changing file extension of not normalised .allValidPairs to avoid conflicts in the next HiC-Pro call

mv "$sample" $(basename "$sample" | sed "s/.allValidPairs/.notnormpairs/")

done

echo "----------------" >> "$parameter3"/log_stats/log_script_HiCPro_"$today".txt

echo " " >> "$parameter3"/log_stats/log_script_HiCPro_"$today".txt

#deleting TEMP .allValidPairs

find "$parameter3"/HiCPro_"$parameter8"_"$today" -type f -name '*_TEMPORARIO.allValidPairs' -exec rm -f {} +

# Continuing with HiC-Pro

echo "--------------------------------------------------------------------" >> "$parameter3"/log_stats/log_script_HiCPro_"$today".txt

echo " " >> "$parameter3"/log_stats/log_script_HiCPro_"$today".txt

echo "-- $(date) -- HiC-Pro step wise from normalisation point (.allValidPairs) to matrix" | tee -a "$parameter3"/log_stats/log_script_HiCPro_"$today".txt

echo " " >> "$parameter3"/log_stats/log_script_HiCPro_"$today".txt

echo "--------------------------------------------------------------------" >> "$parameter3"/log_stats/log_script_HiCPro_"$today".txt

echo " " >> "$parameter3"/log_stats/log_script_HiCPro_"$today".txt

HiC-Pro -i "$parameter3"/hic_results/data/ -o "$parameter3"/HiCPro_"$parameter8"_"$today" -c "$parameter3"/log_stats/"$parameter4" -s build_contact_maps -s ice_norm 2>> "$parameter3"/log_stats/log_script_HiCPro_"$today".txt

fi

#######################

#FURTHER ANALYSIS PART#

#######################

if [ -z "$parameter12" ]; then

echo " " >> "$parameter3"/log_stats/log_script_HiCPro_"$today".txt

echo "-- $(date) -- Just HiC-Pro output / no HOMER, .hic and .cool formats" | tee -a "$parameter3"/log_stats/log_script_HiCPro_"$today".txt

echo " " >> "$parameter3"/log_stats/log_script_HiCPro_"$today".txt

else

echo " " >> "$parameter3"/log_stats/log_script_HiCPro_"$today".txt

echo "-- $(date) -- Transforming HiC-Pro output to HOMER, .hic and .cool formats" | tee -a "$parameter3"/log_stats/log_script_HiCPro_"$today".txt

echo " " >> "$parameter3"/log_stats/log_script_HiCPro_"$today".txt

# Transforming to juicebox format

for jsample in $(find "$parameter3"/HiCPro_"$parameter8"_"$today" -type f -name '*norm.allValidPairs'); do

mkdir -p "$parameter3"/Juicer/$(basename "$jsample" | sed "s/.allValidPairs//")

cd "$parameter3"/Juicer/$(basename "$jsample" | sed "s/.allValidPairs//")

echo "doing .hic"

"$utils_folder"/hicpro2juicebox.sh -i "$jsample" -g "$parameter9" -j "$juicer_folder"/juicer_tools_1.19.02.jar -r "$parameter10"

done &

echo ".hic files ready at $parameter3/Juicer/" &

# Transforming to .cool format

for csample in $(find "$parameter3"/HiCPro_"$parameter8"_"$today" -type f -name '*norm.allValidPairs'); do

mkdir -p "$parameter3"/coolfiles/$(basename "$csample" | sed "s/.allValidPairs//")

cd "$parameter3"/coolfiles/$(basename "$csample" | sed "s/.allValidPairs//")

echo "doing .cool"

"$utils_folder"/hicpro2higlass.sh -i "$csample" -r "$parameter12" -c "$parameter9" -n

done

echo ".cool files ready at $parameter3/coolfiles/" &

# Transforming to HOMER format

for dir in $(ls "$parameter3"/HiCPro_"$parameter8"_"$today"/bowtie_results/bwt2); do

mkdir -p "$parameter3"/Homer/"$dir"

cd "$parameter3"/HiCPro_"$parameter8"_"$today"/bowtie_results/bwt2/"$dir"

R1=$(find "$parameter3"/HiCPro_"$parameter8"_"$today"/bowtie_results/bwt2/"$dir" -type f -name "*_R1_$parameter8.bwt2merged.bam")

samtools merge - $(echo "$R1" | tr '\n' ' ') | samtools view -@ "$parameter6" -h -F 4 - -o "$dir"_R1_homer.sam

echo " "

R2=$(find "$parameter3"/HiCPro_"$parameter8"_"$today"/bowtie_results/bwt2/"$dir" -type f -name "*_R2_$parameter8.bwt2merged.bam")

samtools merge - $(echo "$R2" | tr '\n' ' ') | samtools view -@ "$parameter6" -h -F 4 - -o "$dir"_R2_homer.sam

echo " "

mv *.sam "$parameter3"/Homer/"$dir"

done

for dir in $(ls "$parameter3"/Homer); do

cd "$parameter3"/Homer/"$dir"

echo "doing Homer"

R1=$(find "$parameter3"/Homer/"$dir" -type f -name '*R1_homer.sam')

R2=$(find "$parameter3"/Homer/"$dir" -type f -name '*R2_homer.sam')

makeTagDirectory "$parameter3"/Homer/"$dir" "$R1","$R2" -tbp 1

done

echo "homer dir ready at $parameter3/Homer/"

fi

#remove files with "_TEMPORARIO" tag

find "$parameter3" -name "*_TEMPORARIO*" -exec rm -r {} +

#Adjusting tree structure and *tar..bz2 compressing Intermediate files (*.bam and *.fastq - including Input) that can be later deleted

tar -jcvf "$parameter3"/HiCPro_"$parameter8"_"$today"/bowtie_results.tar.bz2 "$parameter3"/HiCPro_"$parameter8"_"$today"/bowtie_results &

tar -jcvf "$parameter3"/HiCPro_mm10_"$today"/bowtie_results.tar.bz2 "$parameter3"/HiCPro_mm10_"$today"/bowtie_results

#separating "Warnings" from the log file

grep -w '^Warning:' "$parameter3"/log_stats/log_script_HiCPro_"$today".txt >> "$parameter3"/log_stats/log_WARNING.txt

sed -i "/^Warning\:/d" "$parameter3"/log_stats/log_script_HiCPro_"$today".txt

#separating "Warnings" from the log file

cd "$parameter3"/log_stats/

#compiling QC reports

multiqc "$parameter3"/log_stats/ "$parameter3"/HiCPro_"$parameter8"_"$today"/

##################

echo " " >> "$parameter3"/log_stats/log_script_HiCPro_"$today".txt

echo "--------------------------------------------------------------------" >> "$parameter3"/log_stats/log_script_HiCPro_"$today".txt

echo " " >> "$parameter3"/log_stats/log_script_HiCPro_"$today".txt

echo "---END TIME---" >> "$parameter3"/log_stats/log_script_HiCPro_"$today".txt

echo " " >> "$parameter3"/log_stats/log_script_HiCPro_"$today".txt

date >> "$parameter3"/log_stats/log_script_HiCPro_"$today".txt

echo " " >> "$parameter3"/log_stats/log_script_HiCPro_"$today".txt

echo "--------------" >> "$parameter3"/log_stats/log_script_HiCPro_"$today".txt

echo " " >> "$parameter3"/log_stats/log_script_HiCPro_"$today".txt

echo "---FILES & STRUCTURE---" >> "$parameter3"/log_stats/log_script_HiCPro_"$today".txt

echo " " >> "$parameter3"/log_stats/log_script_HiCPro_"$today".txt

tree "$parameter3" >> "$parameter3"/log_stats/log_script_HiCPro_"$today".txt

echo " " >> "$parameter3"/log_stats/log_script_HiCPro_"$today".txt

echo "------" >> "$parameter3"/log_stats/log_script_HiCPro_"$today".txt

echo " " >> "$parameter3"/log_stats/log_script_HiCPro_"$today".txt

echo "Software version" >> "$parameter3"/log_stats/log_script_HiCPro_"$today".txt

echo "------" >> "$parameter3"/log_stats/log_script_HiCPro_"$today".txt

echo "Samtools" >> "$parameter3"/log_stats/log_script_HiCPro_"$today".txt

samtools --version >> "$parameter3"/log_stats/log_script_HiCPro_"$today".txt

echo "Bowtie2" >> "$parameter3"/log_stats/log_script_HiCPro_"$today".txt

bowtie2 --version >> "$parameter3"/log_stats/log_script_HiCPro_"$today".txt

echo "Cutadapt" >> "$parameter3"/log_stats/log_script_HiCPro_"$today".txt

cutadapt --version >> "$parameter3"/log_stats/log_script_HiCPro_"$today".txt

echo "Bedtools" >> "$parameter3"/log_stats/log_script_HiCPro_"$today".txt

bedtools --version >> "$parameter3"/log_stats/log_script_HiCPro_"$today".txt

echo "HiC-Pro" >> "$parameter3"/log_stats/log_script_HiCPro_"$today".txt

HiC-Pro --version >> "$parameter3"/log_stats/log_script_HiCPro_"$today".txt

echo "Homer" >> "$parameter3"/log_stats/log_script_HiCPro_"$today".txt

v4.11.1 >> "$parameter3"/log_stats/log_script_HiCPro_"$today".txt

echo "------ END" >> "$parameter3"/log_stats/log_script_HiCPro_"$today".txt

echo "Please find files in $parameter3"
